# Identification of microRNAs in skeletal muscle associated with lung cancer cachexia

**DOI:** 10.1002/jcsm.12512

**Published:** 2019-12-11

**Authors:** Wouter R.P.H. van de Worp, Annemie M.W.J. Schols, Anne‐Marie C. Dingemans, Céline M.H. Op den Kamp, Juliette H.R.J. Degens, Marco C.J.M. Kelders, Susan Coort, Henry C. Woodruff, Gueorqui Kratassiouk, Annick Harel‐Bellan, Jan Theys, Ardy van Helvoort, Ramon C.J. Langen

**Affiliations:** ^1^ Department of Respiratory Medicine, NUTRIM Maastricht University Medical Center+ Maastricht The Netherlands; ^2^ Department of Bioinformatics—BiGCaT, NUTRIM Maastricht University Medical Center+ Maastricht The Netherlands; ^3^ Department of Precision Medicine, GROW Maastricht University Medical Center+ Maastricht The Netherlands; ^4^ Plateforme ARN interférence, Service de Biologie Intégrative et de Génétique Moléculaire (SBIGeM), I2BC, CEA, CNRS University of Paris‐Saclay Paris France; ^5^ Laboratory of Epigenetics and Cancer, Institut de Hautes Études Scientifiques University of Paris‐Saclay Paris France; ^6^ Nutricia Research, Nutricia Advanced Medical Nutrition Utrecht The Netherlands

**Keywords:** NSCLC, Cancer cachexia, Skeletal muscle, miRNAs, AtromiRs

## Abstract

**Background:**

Cachexia, highly prevalent in patients with non‐small cell lung cancer (NSCLC), impairs quality of life and is associated with reduced tolerance and responsiveness to cancer therapy and decreased survival. MicroRNAs (miRNAs) are small non‐coding RNAs that play a central role in post‐transcriptional gene regulation. Changes in intramuscular levels of miRNAs have been implicated in muscle wasting conditions. Here, we aimed to identify miRNAs that are differentially expressed in skeletal muscle of cachectic lung cancer patients to increase our understanding of cachexia and to allow us to probe their potential as therapeutic targets.

**Methods:**

A total of 754 unique miRNAs were profiled and analysed in vastus lateralis muscle biopsies of newly diagnosed treatment‐naïve NSCLC patients with cachexia (*n* = 8) and age‐matched and sex‐matched healthy controls (*n* = 8). miRNA expression analysis was performed using a TaqMan MicroRNA Array. *In silico* network analysis was performed on all significant differentially expressed miRNAs. Differential expression of the top‐ranked miRNAs was confirmed using reverse transcription–quantitative real‐time PCR in an extended group (*n* = 48) consisting of NSCLC patients with (*n* = 15) and without cachexia (*n* = 11) and healthy controls (*n* = 22). Finally, these miRNAs were subjected to univariate and multivariate Cox proportional hazard analysis using overall survival and treatment‐induced toxicity data obtained during the follow‐up of this group of patients.

**Results:**

We identified 28 significant differentially expressed miRNAs, of which five miRNAs were up‐regulated and 23 were down‐regulated. *In silico* miRNA‐target prediction analysis showed 158 functional gene targets, and pathway analysis identified 22 pathways related to the degenerative or regenerative processes of muscle tissue. Subsequently, the expression of six top‐ranked miRNAs was measured in muscle biopsies of the entire patient group. Five miRNAs were detectable with reverse transcription–quantitative real‐time PCR analysis, and their altered expression (expressed as fold change, FC) was confirmed in muscle of cachectic NSCLC patients compared with healthy control subjects: miR‐424‐5p (FC = 4.5), miR‐424‐3p (FC = 12), miR‐450a‐5p (FC = 8.6), miR‐144‐5p (FC = 0.59), and miR‐451a (FC = 0.57). In non‐cachectic NSCLC patients, only miR‐424‐3p was significantly increased (FC = 5.6) compared with control. Although the statistical support was not sufficient to imply these miRNAs as individual predictors of overall survival or treatment‐induced toxicity, when combined in multivariate analysis, miR‐450‐5p and miR‐451a resulted in a significant stratification between short‐term and long‐term survival.

**Conclusions:**

We identified differentially expressed miRNAs putatively involved in lung cancer cachexia. These findings call for further studies to investigate the causality of these miRNAs in muscle atrophy and the mechanisms underlying their differential expression in lung cancer cachexia.

## Introduction

Lung cancer is the leading cause of cancer deaths worldwide.^1^ The majority of patients (85%) suffer from non‐small cell lung carcinoma (NSCLC) and are predominantly diagnosed at an advanced stage,^2^ and up to 60% suffer from cancer cachexia.^3^ Cachexia is characterized by ongoing loss of body weight, systemic inflammation, anorexia, and pronounced loss of skeletal muscle mass and is identified as an important contributor to poor quality of life and mortality.^3^, ^4^, ^5^ Cachexia also adversely affects cancer therapy as cachectic cancer patients have a decreased response to radiotherapy, chemotherapy, and immunotherapy.^6^, ^7^, ^8^ The proximal pathways associated with muscle wasting in response to inactivity and starvation are well described and similar to those that occur in wasting associated with disease.^9^ However, the triggers and the exact underlying cellular mechanisms of muscle atrophy in lung cancer patients with cachexia remain to be further revealed.

MicroRNAs (miRNAs) are small single‐stranded RNAs of approximately 22 nucleotides long that play a central role in post‐transcriptional gene regulation by either promoting messenger RNA decay or inhibiting translation.^10^ In addition, a number of papers have reported miRNA functioning outside this paradigm (reviewed by Dragomir *et al*.^11^) underlining their importance as regulators of gene expression. MyomiRs, a class of miRNAs exclusively expressed or enriched in striated muscle, have been identified as essential determinants in regulatory networks of myogenesis, muscle fibre‐type composition, muscle growth, and homeostasis.^12^ Several miRNAs have also been shown to play a role in different experimental models of skeletal muscle atrophy, including miR‐18a, miR‐23a, miR‐27a, miR‐29b, miR‐351, miR‐422a, miR‐424(322)‐5p, miR‐542‐5p, and miR‐675.^13^, ^14^, ^15^, ^16^, ^17^, ^18^, ^19^, ^20^, ^21^, ^22^, ^23^, ^24^ AtromiRs have been dubbed as a collective term referring to miRNAs putatively involved in muscle atrophy, regulating processes and signalling pathways that contribute to muscle wasting.^25^, ^26^ However, knowledge of the biological relevance of these miRNAs and their regulation in lung cancer cachexia is lacking.

Until now, extensive profiling of miRNAs related to muscle wasting in human muscle biopsies has been scarce. Changes in intramuscular levels of diverse miRNAs have been implicated in muscle wasting in critical care,^23^ chronic obstructive pulmonary disease (COPD),^24^ amyotrophic lateral sclerosis,^27^, ^28^ and a mixed population of pancreas and colon cancer patients.^29^ However, as the triggers and intracellular mechanisms of muscle atrophy in lung cancer cachexia may partly differ from these other conditions, distinct miRNAs may be involved in lung cancer cachexia. Therefore, we designed the current study to investigate the expression profile of miRNAs in the vastus lateralis muscle of a well‐characterized group of NSCLC patients with cachexia. We aimed to (i) identify differentially expressed miRNAs in lung cancer cachexia and study whether this altered expression profile is already present in lung cancer patients without cachexia; (ii) predict gene targets for the differentially expressed miRNAs and relate these to pathways involved in muscle mass modulation; and (iii) explore the predictive value of these miRNAs for overall survival (OS) and treatment‐induced toxicities.

## Methods

### Study population

The human samples were used from a previously published cross‐sectional study approved by the Medical Ethics Committee of the Maastricht University Medical Centre+ (MEC 06‐2‐015) and conducted according to local ethical guidelines, and all participants provided written informed consent.^30^

Briefly, newly diagnosed patients with advanced stage NSCLC admitted to the Department of Respiratory Medicine of the Maastricht University Medical Centre+ between July 2007 and July 2010 were eligible for participation in the study. Participants were divided into a non‐cachectic and cachectic group according to the definition in the international cachexia consensus.^4^ NSCLC was confirmed by pathological analysis, and tumour stage was determined by using the 6th Tumor–Node–Metastasis International Staging System for Lung Cancer.^31^

To study a representative sample of lung cancer patients but minimize the interference of advanced comorbidities or drugs that could have potential effects on the studied variables, patients with the following characteristics were excluded: Global Initiative for Chronic Obstructive Lung Disease stage IV COPD, Congestive Heart Failure New York Heart Association Stage III–IV, and active infectious disease, as well as patients who were taking hormones or continual oral corticosteroids. Additional exclusion criteria were the presence of other malignant disease and the initiation of antitumor therapy.

Healthy control subjects were recruited through advertisements. It was confirmed that healthy control subjects had no recent body weight loss or any of the diseases or used any of the medications described in the exclusion criteria. Samples from eight NSCLC patients with cachexia and eight healthy controls were used for miRNA array analysis. The extended group included samples from 22 healthy controls, 15 NSCLC patients with cachexia, and 11 NSCLC patients without cachexia.

### Body composition

Body height and waist and hip circumference were measured to the nearest centimetre. Body weight was measured to the nearest 0.1 kg using a standard lance beam scale. Body mass index was calculated as weight/height squared.

Dual‐energy X‐ray absorptiometry (DEXA; DPX‐L, Lunar Radiation Corp., Madison, WI) was used to determine whole‐body composition, including fat mass index, fat‐free mass index (FFMI), and appendicular skeletal muscle index. Appendicular skeletal muscle index was calculated as the lean mass of the extremities divided by body height squared. DEXA measurements were performed in the fasted state.

### Muscle strength

Isokinetic strength of quadriceps muscle was measured by using a Biodex dynamometer (Biodex System Version 3.3). Isokinetic muscle strength testing was performed at an angle of 60° (three repetitions). Muscle strength was defined as the highest muscular force output (peak torque) in Newton metres (N·m).

### Muscle biopsies

Percutaneous needle biopsies of quadriceps muscle (vastus lateralis muscle) were obtained under local anaesthesia using the Bergström technique.^32^ Muscle specimens for biochemical analysis were immediately frozen in liquid nitrogen and stored at −80 °C until further use. Before analysis, muscle biopsies were crushed with a mortar and pestle in liquid nitrogen.

### RNA extraction and microRNA expression analysis

For miRNA expression analysis, TRI Reagent (Sigma‐Aldrich) was used according to the manufacturers' protocol. Muscle specimens (10–30 mg) were homogenized in TRI Reagent by using a Mini Bead Beater (Cole Parmer) sample homogenizer, and total RNA was extracted. A NanoDrop ND‐1000 spectrophotometer (Isogen Life Science) and Agilent 2100 Bioanalyzer (Agilent Technologies) were used to measure the quantity, purity, and integrity of the RNA (Supporting Information, *Table* S1).

### TaqMan® Array Human MicroRNA

Single‐stranded cDNA was synthesized from total RNA samples using the TaqMan MicroRNA Reverse Transcription (RT) Kit (Applied Biosystems) and the Megaplex™ RT primers (Human Pool Set v3.0; Applied Biosystems). A total of 754 miRNAs were profiled from vastus lateralis muscle biopsies (out of a total of 1917 precursors and 2654 mature sequences annotated in miRBase v22).^33^ Briefly, 400 ng of RNA was added to the Megaplex™ RT primer solution containing 0.8 μL of Megaplex™ RT primers (10×), 0.2 μL of deoxynucleotide triphosphate (100 mM), 0.9 μL of MgCl_2_ (25 mM), 0.1 μL of RNase inhibitor (20 U/μL), 1.5 μL of MultiScribe™ Reverse Transcriptase (50 U/μL), 10× RT buffer, and 0.2 μL of nuclease‐free water. RT reactions were carried out according to the manufacturers' protocol.

For TaqMan Array Human MicroRNA, 6 μL of the Megaplex™ RT product was mixed with 2× TaqMan Universal PCR Master Mix (Applied Biosystems) and 444 μL of nuclease‐free water. For each sample, 100 μL of the PCR reaction mix was loaded into each port of the TaqMan Array Cards (Human MicroRNA A + B Set v3.0; Applied Biosystems). The TaqMan Array was carried out on the 7900HT Fast Real‐Time PCR System (Applied Biosystems) according to the manufacturers' protocol. The real‐time expression data were analysed using the 7900 SDS RQ Manager software. The resultant *C*_*q*_ values were exported. GeNorm was used to select the most stable reference gene, *Mamm*U6 (gene stability measure *M*: 0.019 and 0.012, respectively). All *C*_*q*_ values were normalized to *Mamm*U6, using the ΔΔC_t_ method. Cut‐off number of cycles was set as 35. The thresholds used to determine significance of differentially expressed miRNAs were set as *P* < 0.05 and a ΔΔC_t_ ≥ 1 cycle.

### Reverse transcription–quantitative real‐time PCR

A fixed volume of 5 ng/μL of total RNA was used for the RT reaction. First‐strand cDNA synthesis was performed using the miRCURY® LNA® RT Kit according to the manufacturers' protocol (Exiqon). cDNA was diluted (1:80) in nuclease‐free H_2_O and stored at 4 °C, and cDNA stocks were stored at −20 °C. For real‐time PCR amplification, each reaction contained 5 μL of ExiLENT SYBR® Green master mix (Exiqon), 1 μL of miRCURY LNA PCR primer mix (Exiqon), and 4 μL of diluted cDNA template. PCR settings were 95 °C for 10 min, followed by 45 cycles of 95 °C for 10 s and 60 °C for 1 min, carried out on a Roche LightCycler 480 system. Melt curves were made using a gradual increase in temperature of 0.11 °C/s with five acquisitions per second and a temperature range of 60 to 90 °C. The melt curves were examined using the LightCycler 480 software (Roche). PCR efficiency was determined using LinRegPCR software (Supporting Information, *Table* S1). The resultant *C*_*q*_ values were exported and normalized to loading control (UniSp6; Exiqon), using the ΔΔC_t_ method. Cut‐off number of cycles was set as 40. The thresholds used to determine significance of differentially expressed miRNAs were set as *P* < 0.05 and a ΔΔC_t_ ≥ 1 cycle. The primers used are listed in Supporting Information, *Table* S2.

### Network analysis

Cytoscape 3.6.1, a widely adopted network visualization and analysis tool, was used to build a miRNA–gene–pathway network.^34^ The differentially expressed miRNAs were imported into Cytoscape. Using the CyTargetLinker app in Cytoscape,^35^ experimentally validated miRNA–gene interactions from miRTarBase 7.0^36^ were added creating a miRNA‐target interaction network. The targeted genes were reviewed for expression in the skeletal muscle using the Human Protein Atlas.^37^ In addition, an overrepresentation analysis was performed in PathVisio 3.3.0^38^ with the miRNA‐target genes using the human‐curated analysis collection of WikiPathways^39^ and the human gene identifier mapping database (Ensembl 89).^40^ The pathways were then ranked based on a standardized difference score (*Z* score). A pathway was considered involved when the *Z* score >1.96 and permutation *P*‐value <0.05. Finally, the miRNA‐target interaction network was extended with the selected pathways using the human WikiPathways^39^ linkset in the CyTargetLinker app resulting in a miRNA–gene–pathway network. In this network, the miRNA expression levels were visualized. The biological network platform NDEx^41^ was used to share and publish the network.

### Exploration of microRNAs as prognostic factor

Univariate Cox proportional hazard models were constructed using the expression levels of the five differentially expressed miRNAs confirmed in the entire group to assess their potential as prognostic factors for OS and/or treatment‐induced toxicities. OS was defined as the time period between the date of diagnose and the date of death, and the *β*‐coefficient and associated hazard ratio were computed, as well as the significance and the Wald statistics.^42^ Further survival analysis was performed using the median of the survival predictions of multivariate Cox proportional hazard model to stratify the group of patients into distinct survival groups. The logarithms of the expression rates of the miRNAs were considered as continuous variables. Common terminology criteria for adverse events (CTCAE) v5.0 was used to classify treatment‐induced toxicities. CTCAE score of 3 was used as cut‐off point, and random forest classifier models were used to explore cross‐correlation effects between varying combinations of miRNA expression levels on toxicity and the presence of NSCLC, using out‐of‐bag error estimation, and the results are reported as the area under the curve (AUC) of the receiver operator characteristic curve.

### Statistics

Clinical and demographic data of patients and controls are presented as mean ± standard deviation for normally distributed variables and as median (inter‐quartile range) for variables that were not normally distributed. Independent samples *t*‐test and one‐factor analysis of variance were used to calculated differences between continuous variables. *χ* ^2^ test and Fisher's exact test were used to calculate differences between categorical variables. Differences in miRNA expression data were calculated using Student's *t*‐test and one‐factor analysis of variance for normally distributed data or by Mann–Whitney *U* test and Kruskal–Wallis test for non‐parametric data. The log‐rank test for censored survival data was used to calculate the significance of the Cox proportional hazard models. Data were analysed with Statistical Package for the Social Sciences software (SPSS Version 23 for Windows) and R Version 3.4.3.^43^ Significance was set at *P* < 0.05.

## Results

### Clinical characterization of non‐small cell lung cancer patients with and without cachexia and control subjects

Basic characteristics of the study population are detailed in *Table* 1. There was no significant difference between study groups in gender and age. Body weight loss was found to be significantly different between the groups (*P* < 0.001). Non‐cachectic and cachectic patients with NSCLC showed no significant differences in tumour stage or histological subtype. The majority of these patients (non‐cachexia: 64%; cachexia: 73%) suffered from metastatic NSCLC (*Table* 1 and Supporting Information, *Table* S3). At NSCLC diagnosis, non‐cachectic patients showed a mean body weight loss of 1.7% in 6 months before diagnosis, whereas patients with cachexia had a mean body weight loss of 12.7% (*P* < 0.001). Moreover, FFMI was significantly lower in patients with cachexia compared with both healthy controls and non‐cachectic patients (*P* < 0.05), while fat mass index was not different between groups. Muscle strength in patients with cachexia was 47% less than in healthy controls (*P* < 0.05). Non‐cachectic patients showed less pronounced but significantly reduced muscle strength compared with healthy controls (23%, *P* < 0.05).

**Table 1:** Clinical characteristics of the entire study population

		Non‐small cell lung carcinoma (NSCLC)	
	Healthy control (*n* = 22)	Non‐cachexia (*n* = 11)	Cachexia (*n* = 15)
Gender (male/female)	13/9	9/2	8/7
Age (years)	61.4 ± 7.0	63.3 ± 10.3	58.9 ± 7.8
Height (m)	1.73 ± 0.10	1.76 ± 0.06	1.72 ± 0.10
Body weight loss (kg)	0 ± 0	1.3 ± 1.1^a^	9.7 ± 3.9^b^^,^ c
Body weight loss (%)	0 ± 0	1.7 ± 1.3^a^	12.7 ± 4.9^b^^,^ c
Body mass index (BMI) (kg/m^2^)	24.1 ± 3.3	25.3 ± 3.4	23.1 ± 4.9
Fat mass index (FMI) (kg/m^2^)	6.5 ± 2.5	7.6 ± 2.8	6.8 ± 3.1
Fat‐free mass index (FFMI) (kg/m^2^)	18.4 ± 2.2	18.2 ± 1.8	16.6 ± 2.8^b^
Appendicular skeletal muscle index (kg/m^2^)	7.7 ± 1.0	7.1 ± 0.84	6.2 ± 0.97^b^^,^ c
IL‐6 (pg/mL)	42.6 (34.79)	55.7 (44.82)	130.6 (81.199)^b^^,^ c
CRP (mg/L)	1.0 (0.5. 1.5)	9.5 (5, 40)^a^	49.5 (25.86)^b^^,^ c
Disease stage (IIIB/IV)	—	4/7	4/11
Histology (adeno/squamous cell)	—	8/3	8/7
Peak torque flexion 60° (N·m)	73.9 ± 20.8	57.3 ± 19.7^a^	37.4 ± 16.7^b^^,^ c
Peak torque extension 60° (N·m)	120.0 ± 31.2	92.1 ± 27.8^a^	63.6 ± 28.3^b^^,^ c

CRP, C‐reactive protein; IL, interleukin.

Cachexia was defined as body weight loss of >5% in the past 6 months or body weight loss of >2% in combination with either a BMI < 20 kg/m^2^ or an appendicular skeletal muscle index consistent with sarcopenia determined by DEXA (male <7.26 kg/m^2^; female <5.45 kg/m^2^). Data are presented as mean ± standard deviation for normally distributed variables and as median (inter‐quartile range) for variables that were not normally distributed. Significance was calculated by one‐factor analysis of variance and least significant difference *post hoc* testing for normally distributed variables and by Mann–Whitney *U* test for variables that were not normally distributed.

a *P* < 0.05 (non‐cachexia compared with control subjects)

b *P* < 0.05 (cachexia compared with control subjects).

c *P* < 0.05 (non‐cachexia compared with cachexia).

### Identification of differentially expressed microRNAs in non‐small cell lung cancer cachexia using TaqMan Array Human MicroRNA

**Figure 1:**
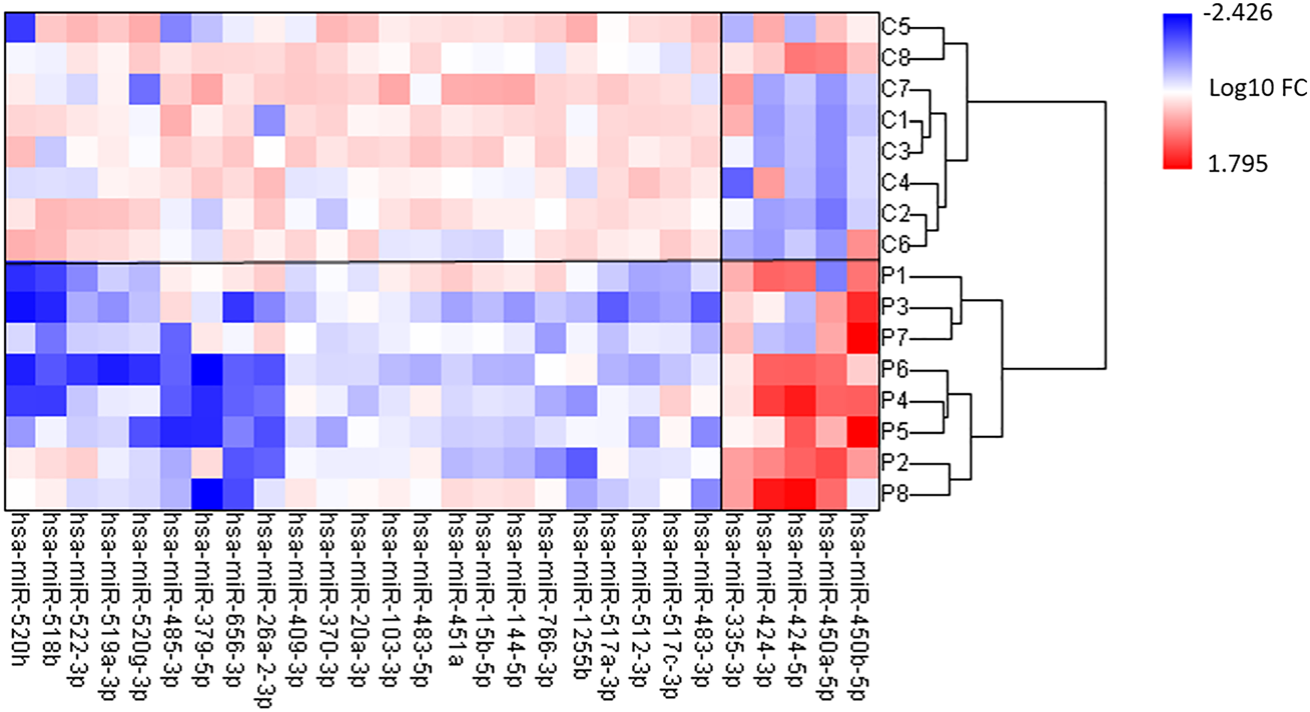
Heatmap showing the 28 microRNAs (miRNAs) found to be differentially expressed in skeletal muscle of non‐small cell lung cancer (NSCLC) patients with cachexia (P, *n* = 8) in comparison with age‐matched healthy controls (C, *n* = 8). The expression of miRNAs was measured by TaqMan® Array Human MicroRNA in vastus lateralis muscle biopsies as described in the Methods section. Red: up‐regulated miRNAs; blue: down‐regulated miRNAs. Only those miRNAs with a *P* < 0.05 and fold change (FC) ≥ 2 are shown (two‐tailed *t*‐test). The numbers on the legend are log_10_‐transformed values. Hierarchical cluster analysis of all subjects using Ward's method was conducted, resulting in a separation into cachectic NSCLC patients and healthy controls, respectively.

Muscle biopsies from eight NSCLC patients with cachexia and eight gender‐matched and age‐matched healthy controls were subjected to TaqMan Array to identify miRNAs associated with lung cancer cachexia. Clinical and demographic data of the patients and controls are detailed in Supporting Information, *Table* S4. A total of 754 miRNAs were profiled from vastus lateralis muscle biopsies. Twenty‐eight miRNAs were found to be differentially expressed in cachectic NSCLC patients compared with healthy controls (*Figure* 1 and Supporting Information, *Table* S5). A total of 5 miRNAs were up‐regulated [fold change (FC) ≥ 2 and a *P* < 0.05]. Remarkably, four of them (miR‐450a‐5p, miR‐450b‐5p, miR‐424‐5p, and miR‐424‐3p) belong to the same miRNA cluster. The majority of the differentially expressed miRNAs (82%) were down‐regulated (FC ≤ 0.5 and a *P* < 0.05). Hierarchical cluster analysis of all subjects using Ward's method was conducted (*Figure* 1), resulting in a separation into two equal clusters consisting of cachectic NSCLC patients and healthy controls, respectively. These findings suggest a disease‐related difference in miRNA expression profile between NSCLC patients with cachexia and healthy controls.

### Predicted microRNA–gene–pathway network

Differentially expressed miRNAs may contribute to regulation of muscle wasting in lung cancer cachexia, which implies that relevant biochemical pathways in the muscle are targeted. To identify potentially involved pathways linked to these miRNAs, miRTarBase was utilized for miRNA‐target prediction based on experimentally validated targets. This *in silico* analysis revealed that 3215 miRNA‐target genes and 5164 miRNA‐target interactions were found. Only miRNA‐target genes with a confirmed functional interaction were selected. For 24 out of 28 differentially expressed miRNAs, functional gene targets were present in miRTarBase. Analysis showed 158 genes to be targeted, some of which by more than one of the miRNAs. Of these target genes, 114 are functionally expressed in the skeletal muscle, and several of them have already been studied in the context of cancer cachexia or other muscle wasting disorders (Supporting Information, *Table* S6).

**Figure 2:**
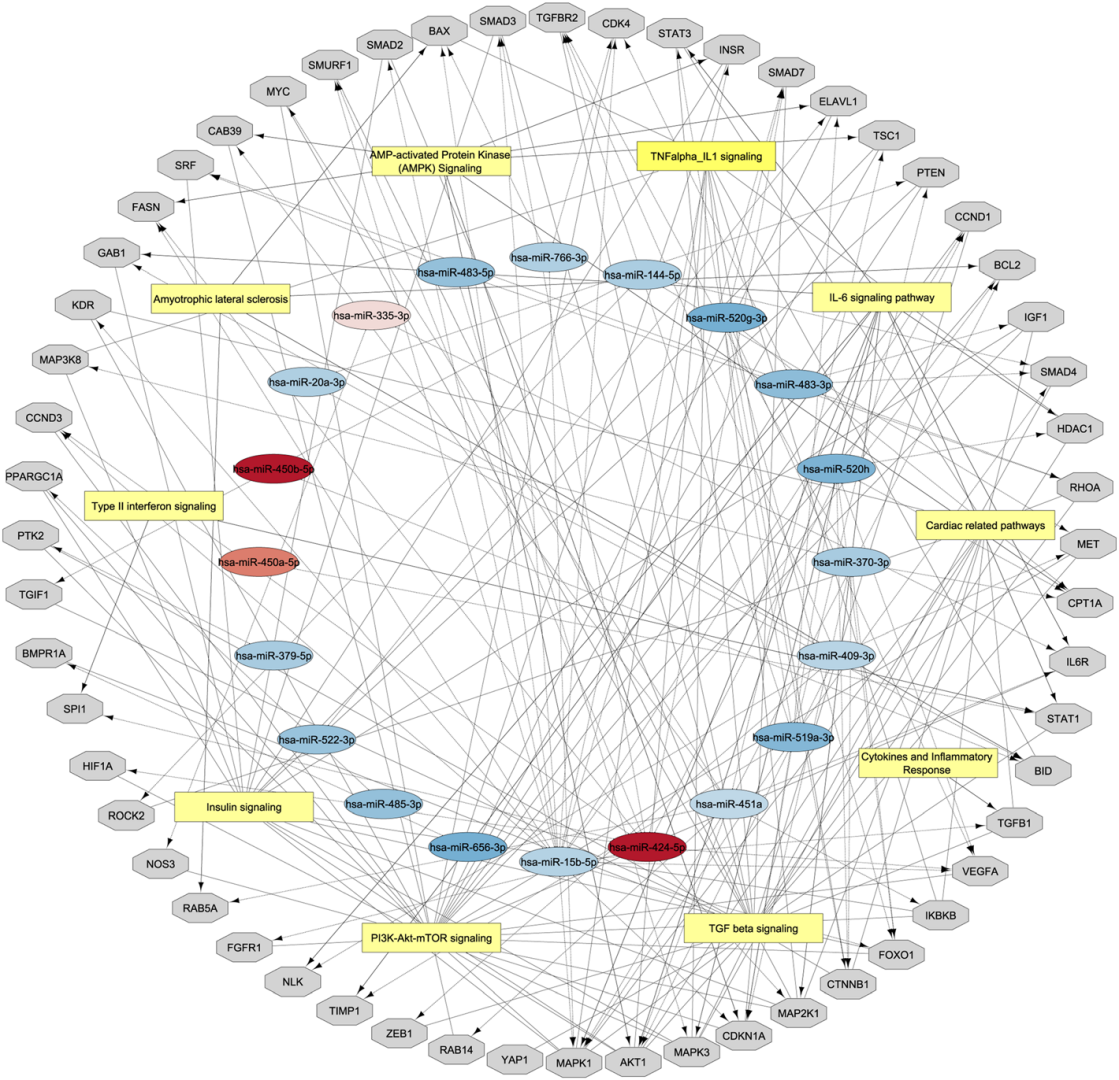
microRNA (miRNA)–gene–pathway network based on miRTarBase and WikiPathways as described in the Methods section. Red: up‐regulated miRNAs; blue: down‐regulated miRNAs; grey: potential gene targets in the skeletal muscle; and yellow: most significantly targeted pathways. Cytoscape was used to visualize the network. The network is available via NDEx. The miRNAs and associated target genes within each pathway are included in Supporting Information, *Tables* S7a and S7b.

Using the 158 genes, an overrepresentation analysis was performed in PathVisio using the human‐curated analysis collection of WikiPathways. Out of 463 pathways, 181 met the criteria (*Z* score >1.96 and permutation *P*‐value <0.05). A selection of pathways considered relevant based on existing literature implying their involvement in muscle tissue regenerative and degenerative processes was added into the network (*Figure* 2 and Supporting Information, *Tables* S7a and S7b) and included interleukin‐6, transforming growth factor‐β, insulin, PI3K–Akt, and tumour necrosis factor‐α signalling pathways. A table of all significantly targeted pathways is available in Supporting Information, *Table* S8.

### Confirmation and comparison of the top‐ranked differentially expressed microRNAs in the entire study population

**Figure 3:**
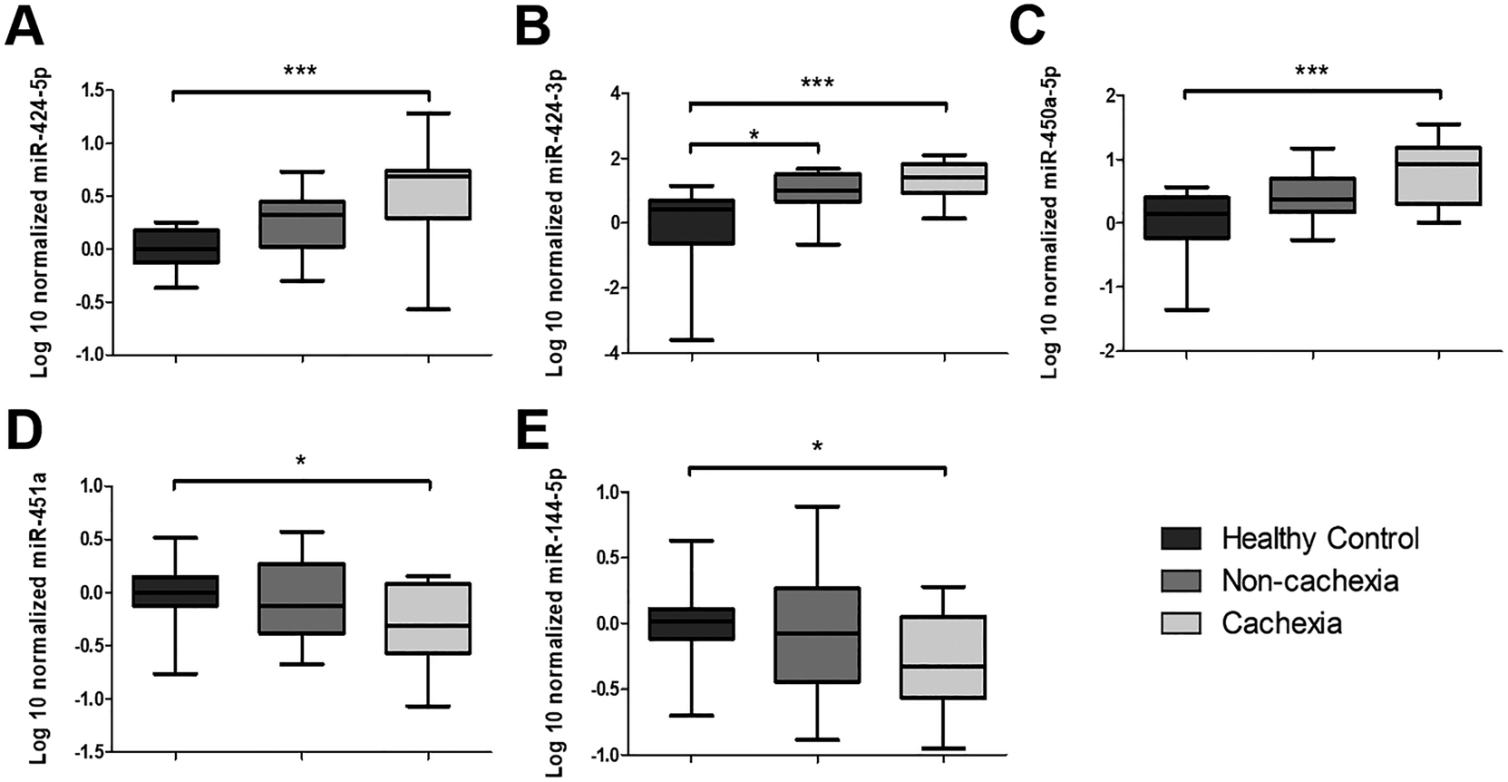
Box‐and‐whisker plots showing that the expression of (A) miR‐424‐5p, (B) miR‐424‐3p, and (C) miR‐450a‐5p is increased and the expression of (D) miR‐451a and (E) miR‐144‐5p is decreased in non‐small cell lung cancer (NSCLC) patients with cachexia compared with control. The expression of the microRNAs was determined by quantitative PCR in vastus lateralis muscle biopsies from the extended cohort of cachectic (*n* = 15) and non‐cachectic (*n* = 11) NSCLC patients and healthy controls (*n* = 22) as described in the Methods section. The box‐and‐whisker plot shows median and min to max. ^*^ *P* < 0.05 and ^***^ *P* < 0.001, Kruskal–Wallis test and Dunn *post hoc* testing.

To assess whether this miRNA expression pattern is specific to lung cancer, or lung cancer cachexia, a selection of six top‐ranked miRNAs was measured in the muscle biopsies of the entire group, consisting of treatment‐naïve NSCLC patients with cachexia (*n* = 15) and without cachexia (*n* = 11) and healthy controls (*n* = 22). The selection of miRNAs was based on their magnitude of differences in expression and postulated relevance of these miRNAs in muscle mass modulation. The six highest‐ranked miRNAs (four up‐regulated and two down‐regulated) were measured by reverse transcription–quantitative real‐time PCR. MiR‐450b‐5p expression levels could not reliably be detected (*C*_*q*_ > 40) and were omitted from further analyses. Five miRNAs showed a significant difference between cachectic NSCLC patients and healthy controls (*Figure* 3). MiR‐424‐5p, miR‐424‐3p, and miR‐450a were 4.5‐fold, 12‐fold, and 7.7‐fold increased, respectively (*Figure* 3A–3C). MiR‐451a and miR‐144‐5p were 1.75‐fold and 1.69‐fold decreased, respectively (*Figure* 3D and 3E). Only miR‐424‐3p was significantly increased in non‐cachectic patients compared with healthy controls (*Figure* 3B). No gender‐related differences were observed in miRNA expression within the groups (data not shown). Overall, these data confirm the differential expression of five miRNAs in skeletal muscle of NSCLC patients with cachexia compared with controls and reveal intermittent changes in non‐cachectic patients for induced miRNAs only.

Subsequently, a machine learning approach was used to explore cross‐correlation effects between varying combinations of miRNA expression levels and the presence of cachexia. A random forest classifier to differentiate between NSCLC patients and healthy individuals using miRNA expression as predictors for cachexia yielded results significantly different from random chance (AUC = 0.5) for the combination of miR‐424‐3p and miR‐450a‐5p yielding an AUC = 0.79 (95% confidence interval 0.6–0.9) and addition of miR‐144‐5p increasing the AUC to 0.85 (95% confidence interval 0.7–0.9). Further addition of differentially expressed miRNAs did not significantly increase the AUC. These data further confirm the correlation of these miRNAs with cachexia and implicate miR‐424‐3p, miR‐450a‐5p, and miR‐144‐5p as putative AtromiRs.

### microRNAs as potential independent prognostic and predictive factors?

**Figure 4:**
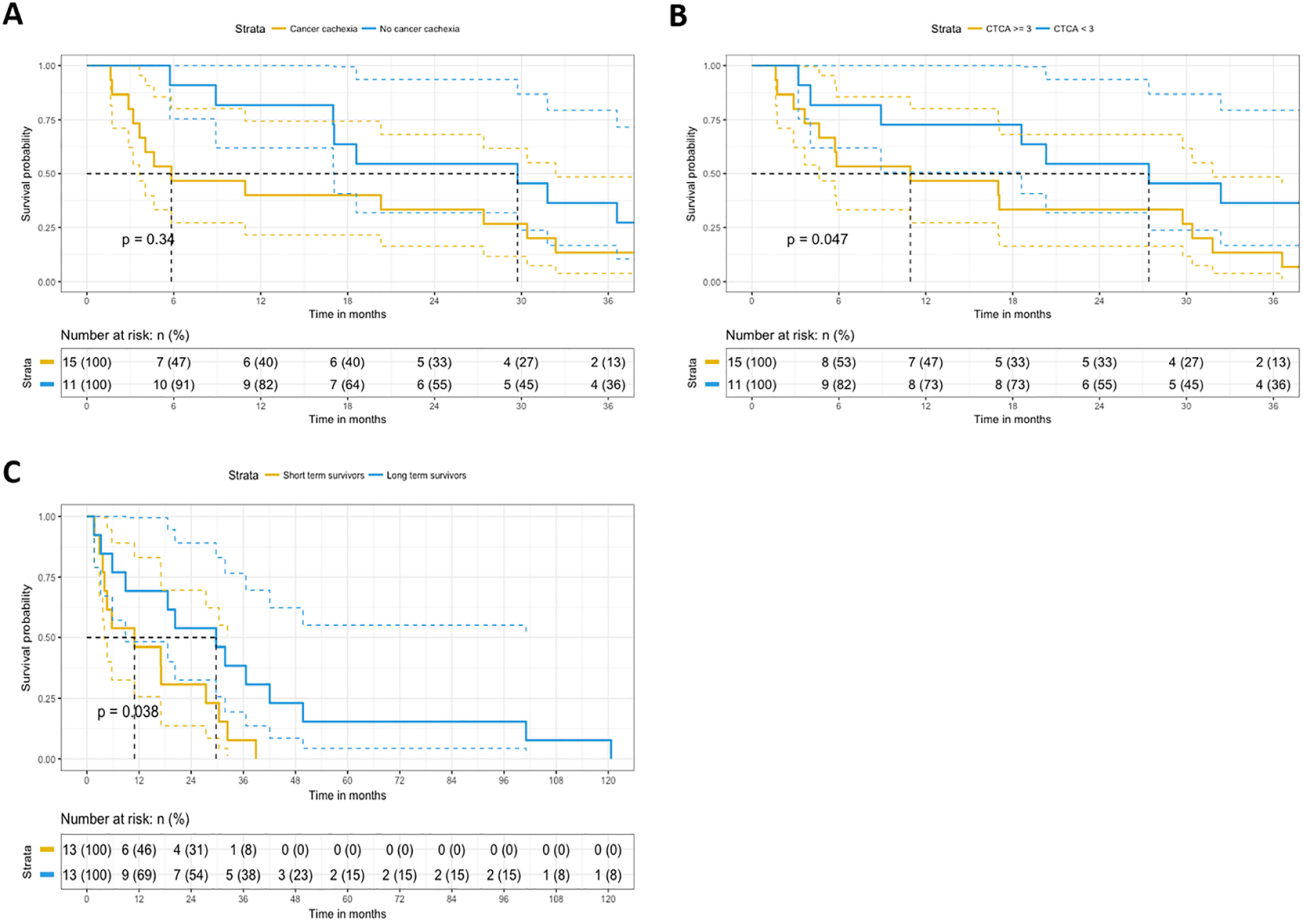
Kaplan–Meier plots for cachexia, treatment‐induced toxicity, and microRNA (miRNA) expression. (A) Kaplan–Meier plot was constructed to assess the survival function of non‐small cell lung cancer (NSCLC) patients with cachexia (*n* = 15) vs. without cachexia (*n* = 11). Cachectic NSCLC patients had a shorter overall survival (OS) when compared with non‐cachectic NSCLC patients. The log‐rank *P*‐value was not significant. (B) Kaplan–Meier plot was constructed to assess the survival function of NSCLC patients with a CTCAE ≥ 3 vs. NSCLC patients with a CTCAE < 3. Patients received either chemotherapy or a combination of chemotherapy and radiotherapy. NSCLC patients with a CTCAE ≥ 3 had a shorter OS compared with NSCLC patients with a CTCAE < 3. The log‐rank *P*‐value was not significant. (C) Kaplan–Meier plot was constructed to assess the survival function of miRNA expression in NSCLC. Multivariate analysis using the miRNAs with the lowest *P*‐value and highest hazard ratios (miR‐450a‐5p and miR‐451a) in linear combination yield a significant stratification into two distinct survival groups (short‐term and long‐term survival).

Survival data were available of this well‐characterized NSCLC study population and showed that patients with cachexia have a decreased 3 year OS rates (*Figure* 4A). Moreover, of the patients with a high treatment‐induced toxicity score (CTCAE ≥ 3), cachectic patients had a significantly lower OS compared with non‐cachectic patients (*Figure* 4B). To test whether the expression levels of miRNAs are potential prognostic factors for OS and/or treatment‐induced toxicities, the five differentially expressed miRNAs confirmed in the entire group were subjected to univariate Cox proportional hazard analysis. Univariate Cox proportional hazard results for OS showed no significant correlation between OS and miRNA expression. Moreover, miR‐450a‐5p achieved a high hazard ratio of 2.3, which, although not significantly different (*P* = 0.09; *Table* 2), may indicate a potential association between the miR‐450a‐5p expression and OS. Multivariate analysis using the miRNAs with the lowest *P*‐value and highest hazard ratios (miR‐450a‐5p and miR‐451a) in linear combination yield a significant stratification into two distinct survival groups (*P* = 0.038; *Figure* 4C). In linear regression analysis to find correlations with the CTCAE scale revealed, none of the miRNAs showed up as a potential classifier for treatment‐induced toxicities.

**Table 2:** Univariate Cox proportional hazard results for overall survival

miRNAs	*β*	HR (95% CI for HR)	Wald test	*P*‐value
hsa‐miR‐424‐5p	−0.09	0.9 (0.3–3.0)	0.0	0.89
hsa‐miR‐451a	−0.67	0.5 (0.2–1.7)	1.2	0.27
hsa‐miR‐144‐5p	−0.46	0.6 (0.2–1.8)	0.7	0.40
hsa‐miR‐424‐3p	−0.01	1.0 (0.5–1.9)	0.0	0.98
hsa‐miR‐450a‐5p	0.82	2.3 (0.9–5.8)	2.9	0.09

CI, confidence interval; HR, hazard ratio; miRNAs, microRNAs.

Five top‐ranked miRNAs were subjected to univariate proportional hazard model. No significant correlation between overall survival and miRNA expression was found.

## Discussion

Cancer cachexia treatment is an unmet medical need, and a better understanding of the underlying mechanisms is essential to develop effective interventions. In this study, we identified 28 differentially expressed miRNAs in the skeletal muscle of cachectic lung cancer patients. Subsequent validation of six top‐ranked miRNAs revealed that differential expression of four miRNAs is unique to patients with cachexia. In addition, using a random forest model trained on three differentially expressed miRNAs allowed for the classification between healthy individuals and NSCLC patients with cachexia. Extensive target prediction and pathway analyses linked the differentially expressed miRNAs to processes involved in muscle mass modulation. Finally, using Cox proportional hazard models including the top two performing miRNAs, significant stratification between short‐term and long‐term survival was achieved.

Of 28 differentially expressed miRNAs, five miRNAs are overexpressed in the vastus lateralis of NSCLC patients with cachexia compared with healthy controls. Remarkably, four of these miRNAs belong to the same cluster, the H19X‐encoded miR‐424/miR‐503 cluster. This miRNA cluster includes six miRNAs (miR‐424, miR‐503, miR‐542, miR‐450a‐1, miR‐450a‐2, and miR‐450b) and has postulated roles in cell differentiation, proliferation, plasticity, and metabolism, extensively reviewed by Wang *et al*..^44^ In accordance to our data, members of this miRNA cluster were previously found to be increased in the skeletal muscle of patients with other muscle wasting diseases: in patients with amyotrophic lateral sclerosis, the expression levels of miR‐425, miR‐450b, miR‐503, and miR‐542 were increased.^27^, ^28^ In addition, the expression levels of miR‐424‐5p/3p, miR‐450a, and miR‐542‐5p/3p were found to be increased in COPD patients with low FFMI, and patients with intensive care unit acquired weakness.^22^, ^23^, ^24^

The expression of the miR‐451a cluster, including miR‐451a and miR‐144, is significantly decreased in the skeletal muscle of cachectic NSCLC patients. The miR‐451a cluster is located on chromosome 17,^45^ and is widely dysregulated in numerous human cancers in which it may play an important role in proliferation, migration and invasion of cells.^46^, ^47^ However, knowledge of the mechanism of action of the miR‐451a cluster in skeletal muscle is currently limited. Coherent with our data, miR‐144 was significantly down‐regulated in the skeletal muscle of chronic kidney disease patients and COPD patients with a low FFMI.^23^, ^48^ Expression of the other member of the miR‐451a cluster, miR‐451a, was significantly increased in the skeletal muscle of powerlifters.^49^ D'Souza *et al*. postulated a supportive role of miR‐451a in myogenesis, and its decreased expression observed in our study may relate to impairments in the myogenesis process, which have been implicated in cancer cachexia.^30^, ^50^, ^51^

Another miRNA cluster that is abundantly represented by the differentially expressed miRNAs is the C19 miRNA cluster (C19MC). The expression of nine miRNAs from the C19MC is suppressed in the vastus lateralis of NSCLC patients with cachexia. C19MC is one of the largest miRNA clusters in human genome.^52^, ^53^ The miRNAs of the C19MC are expressed from the paternal chromosome and promote pluripotent stem cell phenotype,^52^ and their activity towards promoting stem cell survival has been implicated in muscle mass maintenance.^24^ In general, paternally imprinted genes promote growth, which is consistent with our data revealing decreased expression of these miRNAs in cachexia. Similar to our findings, the expression of multiple members from the C19MC is suppressed in the skeletal muscle of COPD patients with low FFMI.^24^ In that study, an association of C19MC expression with FFMI was observed, which was restricted to male patients with severe COPD and absent in female patients. Although a potential interaction between sex and miRNA expression on the cachexia could not be investigated in our relatively small group, sexual dimorphism of skeletal muscle regulation has been reported^54^ and should be subject of future study.

Altogether, these findings show changes in expression of overlapping miRNA in different wasting disorders. Although the number of human miRNA profiling studies related to muscle wasting is limited, these miRNA clusters seem promising targets in the search of miRNAs involved in muscle atrophy.

The current study was not designed to dissect molecular mechanisms of miRNA involvement in cachexia. However, *in silico* network analysis provided relevant information about the potential contribution of these miRNAs in lung cancer cachexia. Using miRTarBase, we identified 158 experimentally validated genes that are potentially targeted by the miRNAs. Among these genes, 114 are functionally expressed in the skeletal muscle and are involved in pathways contributing to muscle degenerative and regenerative processes. The overrepresented pathways that have been implicated in muscle mass regulation include interleukin‐6 signalling pathway, transforming growth factor‐β signalling pathway, tumour necrosis factor‐α signalling pathway, and insulin and PI3K–Akt signalling pathway, reviewed in.^55^, ^56^, ^57^ The involvement of these pathways in muscle atrophy has been established in various experimental models, and their detection as targets of the differentially expressed miRNAs identified in this study, for the first time, implies their potential involvement in lung cancer cachexia. Furthermore, the identification of pathways relevant to muscle mass regulation using this approach confirms our strategy to combine miRNA expression and network analyses and emphasizes the urgency to explore the causal involvement of differential expression of these miRNAs in cancer cachexia.

The data obtained from the miRNA screening and *in silico* network analysis suggest a potential role for the differentially expressed miRNAs in the pathology of lung cancer cachexia. However, the experimental set‐up applied in the screening approach did not allow us to discriminate between lung cancer and lung cancer cachexia‐specific miRNAs. For that reason, a selection of top‐ranked miRNAs was measured in the entire study population consisting of healthy controls and NSCLC patients with and without cachexia. Consistent with the results of the TaqMan Array, miR‐424‐5p, miR424‐3p, and miR‐450a‐5p were significantly increased, and miR‐451a and miR‐144‐5p were significantly decreased in NSCLC patients with cachexia compared with healthy controls. Except for miR‐424‐3p, the three up‐regulated miRNAs showed a non‐significant but intermediate expression in NSCLC patients without cachexia. These intermediate expression levels might reflect a pre‐cachectic stage in some of NSCLC patients classified as non‐cachectic. Although the anthropomorphic data were not available beyond the cross‐sectional comparison at diagnosis, up to 60% of NSCLC patients may eventually develop cachexia,^3^ supporting the possibility that a portion of the patients were pre‐cachectic. Consequently, miR‐424‐5p, miR‐424‐3p, and miR‐450a‐5p may play a role in the development of cachexia in lung cancer. In contrast, down‐regulated miRNAs did not display intermediate expression levels in non‐cachectic lung cancer patients. As decreased expression of miR‐451a and miR‐144‐5p seems to be lung cancer cachexia specific, this may reflect adaptive changes of these miRNAs to the cachectic state. For the prediction of cachexia, when differentiating between NSCLC patients and healthy controls, we found results significantly different from random chance. These data are promising; however, given the low number of patients and relatively high number of variables (five per patient), these results are exploratory and need to be validated externally in a larger cohort.

Body weight loss has a detrimental impact on survival in NSCLC. In different study populations, it has been shown that increased body weight loss results in shorter OS.^6^, ^58^, ^59^, ^60^ Furthermore, it has been shown that low muscle mass is associated with increased treatment‐induced toxicities.^61^ Consistent with these findings, our data show that NSCLC patients with cachexia have a shorter 3 year survival time compared with NSCLC patients without cachexia. Moreover, we show that OS is more affected by treatment‐induced toxicity in cachectic compared with non‐cachectic patients. In this study, we tested the potential of miRNAs as independent prognostic factor for OS and/or treatment‐induced toxicities using a univariate analysis. In addition, using a multivariate Cox proportional hazard model including the top 2 performing miRNAs from univariate analysis, significant stratification of the patient population into short‐term and long‐term survival was achieved. Extending this approach in a larger cohort of lung cancer patients is required and may yield predictive or prognostic miRNA signatures, as was shown for OS in abdominal cancer.^29^

## Conclusions

We identified 28 differentially expressed miRNAs in the skeletal muscle of lung cancer patients with cachexia, including five top‐ranked miRNAs with high potential relevance to muscle atrophy, as demonstrated using gene target/pathway analyses. Further investigation of these miRNAs to elucidate their regulation and mode of action in muscle atrophy will contribute to our understanding of cachexia and allow us to probe their potential as therapeutic targets.

## Conflict of interest

W.R.P.H.v.d.W., A.M.W.J.S., A‐M.C.D., C.M.H.O.d.K., J.H.R.J.D., M.C.J.M.K., S.C., H.C.W., G.K., A.H‐B., J.T., and R.C.J.L. do not have any conflicts of interest. A.v.H. is employed by Nutricia Research.

## Funding

This study was in part supported by ZonMW Project No. 92003564.

## Supporting information


**Table S1**. RNA quality and PCR efficiency for all samples.
**Table S2**. miRCURY LNA miRNA PCR assay primers
**Table S3**. Tumor stage and site of metastasis.
**Table S4**. Clinical and demographic data of NSCC patients with cachexia and controls subjected to TaqMan® miRNA array
**Table S5**. significantly differentially expressed miRNAs in cachectic NSCLC patients compared to healthy controls.
**Table S6**. Genes significantly targeted by the differentially expressed miRNAs
**Table S7a**. MiRNA‐gene interactions included in the network.
**Table S7b** Pathway‐gene interactions included in the network.
**Table S8**. Pathways significantly targeted by the differentially expressed miRNAs
