# Supplementary material for: Identification of microRNAs in skeletal muscle associated with lung cancer cachexia
Source: J Cachexia Sarcopenia Muscle. 2019 Dec 11;11(2):452–63. doi: 10.1002/jcsm.12512 (PMC7113505; doi:10.1002/jcsm.12512)
Supplement: Supplementary file 1 — Table S1. RNA quality and PCR efficiency for all samples. Table S2. miRCURY LNA miRNA PCR assay primers Table S3. Tumor stage and site of metastasis. Table S4. Clinical and demographic data of NSCC patients with cachexia and controls subjected to TaqMan® miRNA array Table S5. significantly differentially expressed miRNAs in cachectic NSCLC patients compared to healthy controls. Table S6. Genes significantly targeted by the differentially expressed miRNAs Table S7a. MiRNA‐gene interactions included in the network. Table S7b Pathway‐gene interactions included in the network. Table S8. Pathways significantly targeted by the differentially expressed miRNAs [file JCSM-11-452-s001.docx]

# Supporting Information

**Supplemental table 1: RNA quality and PCR efficiency for all samples.**

|  | **RNA quality** | **Individual PCR efficiency** | | | | | **GeNorm** |
| --- | --- | --- | --- | --- | --- | --- | --- |
| **Sample** | **A260/280** | **miR-424-5p** | **miR-451a** | **miR-144-5p** | **miR-424-3p** | **miR-450a** | **mRNA** |
| 1_1 | 1.99 | 1.922 | 1.885 | 1.915 | 1.835 | 1.915 | 0.8598 |
| 2_1 | 1.89 | 1.882 | 1.965 | 1.939 | 1.825 | 1.890 | 0.7994 |
| 3_1 | 2.05 | 1.964 | 1.887 | 1.848 | 1.856 | 1.904 | 1.1624 |
| 4_1 | 1.96 | 1.961 | 1.923 | 1.907 | 1.853 | 1.883 | 0.9871 |
| 5_1 | 2.01 | 1.865 | 1.925 | 1.911 | 1.873 | 1.939 | 0.8769 |
| 6_1 | 1.99 | 1.916 | 1.996 | 1.848 | 1.832 | 1.899 | 1.0102 |
| 7_1 | 2.01 | 1.830 | 1.904 | 1.952 | 1.857 | 1.913 | 0.6178 |
| 8_1 | 2.01 | 1.859 | 1.897 | 1.992 | 1.915 | 1.910 | 1.2178 |
| 9_1 | 1.97 | 1.890 | 1.888 | 1.802 | 1.888 | 1.909 | 1.0354 |
| 10_1 | 1.99 | 1.870 | 1.934 | 1.877 | 1.845 | 1.861 | 1.0114 |
| 11_1 | 2.01 | 1.836 | 1.887 | 1.932 | 1.826 | 1.891 | 0.7607 |
| 12_1 | 2.02 | 1.891 | 1.864 | 1.980 | 1.863 | 1.912 | 0.6387 |
| 13_1 | 2.02 | 1.825 | 1.888 | 1.809 | 1.871 | 1.913 | 1.3639 |
| 14_1 | 2.08 | 1.767 | 1.947 | 2.038 | 1.869 | 1.915 | 0.8679 |
| 15_1 | 2 | 1.942 | 1.941 | 1.839 | 1.852 | 1.938 | 1.0712 |
| 16_1 | 2.02 | 1.883 | 1.917 | 1.849 | 1.904 | 1.932 | 0.9908 |
| 17_1 | 1.96 | 1.883 | 1.861 | 1.807 | 1.862 | 1.949 | 0.7364 |
| 18_1 | 2.03 | 1.897 | 1.896 | 1.844 | 1.866 | 1.929 | 0.835 |
| 19_1 | 1.99 | 1.849 | 1.905 | 1.882 | 1.867 | 1.952 | 1.5495 |
| 20_1 | 2.03 | 1.824 | 1.874 | 1.993 | 1.845 | 1.883 | 1.846 |
| 21_1 | 2 | 1.891 | 1.944 | 1.808 | 1.880 | 1.915 | 1.0113 |
| 22_1 | 2.02 | 1.916 | 1.946 | 1.894 | 1.877 | 1.924 | 1.6542 |
| 23_1 | 2.09 | 1.778 | 1.839 | 1.841 | 1.860 | 1.867 | 1.1407 |
| 24_1 | 2.03 | 1.860 | 1.879 | 1.887 | 1.842 | 1.879 | 1.4856 |
| 25_1 | 2.03 | 1.919 | 1.938 | 1.900 | 1.844 | 1.759 | 0.8038 |
| 26_1 | 2.03 | 1.854 | 1.881 | 1.838 | 1.889 | 1.897 | 1.406 |
| 1_2 | 2.09 | 1.815 | 1.940 | 1.921 | 1.000 | 1.872 | 1.0414 |
| 2_2 | 2.01 | 1.912 | 1.933 | 1.787 | 1.866 | 1.911 | 1.3722 |
| 3_2 | 2.01 | 1.832 | 1.888 | 1.786 | 1.879 | 1.872 | 1.1855 |
| 4_2 | 1.99 | 1.846 | 1.889 | 1.972 | 1.859 | 1.895 | 1.0323 |
| 5_2 | 1.88 | 1.810 | 1.949 | 1.830 | 1.866 | 1.882 | 1.1018 |
| 6_2 | 1.96 | 1.828 | 1.951 | 1.919 | 1.841 | 1.865 | 0.6143 |
| 7_2 | 2 | 1.852 | 1.936 | 1.878 | 1.817 | 1.000 | 1.318 |
| 8_2 | 2.12 | 1.867 | 1.867 | 1.984 | 1.841 | 1.879 | 0.6554 |
| 9_2 | 2.01 | 1.879 | 1.892 | 1.914 | 1.887 | 1.912 | 0.9725 |
| 10_2 | 2 | 1.824 | 1.900 | 1.917 | 1.862 | 1.871 | 0.8296 |
| 11_2 | 2.07 | 1.784 | 1.856 | 1.843 | 1.817 | 1.000 | 1.1669 |
| 12_2 | 1.98 | 1.991 | 1.915 | 1.857 | 1.000 | 1.918 | 0.8791 |
| 13_2 | 2 | 1.787 | 1.888 | 1.788 | 1.871 | 1.890 | 0.766 |
| 14_2 | 2.02 | 1.870 | 1.946 | 1.886 | 1.858 | 1.927 | 0.875 |
| 15_2 | 2.02 | 1.966 | 1.888 | 1.954 | 1.854 | 1.892 | 0.9135 |
| 16_2 | 2.02 | 1.844 | 1.911 | 1.867 | 1.857 | 1.891 | 0.8824 |
| 17_2 | 2.02 | 1.850 | 1.893 | 1.958 | 1.853 | 1.904 | 0.9484 |
| 18_2 | 2.09 | 1.879 | 1.851 | 1.920 | 1.786 | 1.901 | 1.7224 |
| 19_2 | 2.09 | 1.814 | 1.887 | 1.820 | 1.553 | 1.917 | 1.0973 |
| 20_2 | 2.06 | 1.898 | 1.937 | 2.032 | 1.859 | 1.874 | 0.8025 |
| 21_2 | 2.07 | 1.857 | 1.899 | 2.029 | 1.871 | 1.895 | 1.0101 |
| 22_2 | 1.96 | 1.940 | 1.887 | 1.971 | 1.820 | 1.902 | 0.9308 |

***RNA quality (A260/280) was assessed using the ND-1000 Nanodrop spectrophotometer. PCR efficiency was determined for each sample and primer using LinRegPCR software. GeNorm. a gene expression normalization factor was calculated for each sample based on the geometric mean of 4 reference genes (Cyclophilin A. beta-Actin. RPL13A and RPLPO)***

**Supplemental table 2: miRCURY LNA miRNA PCR assay primers**

| **Product name** | **Target sequence 5’-3’** | **Cat. no.** | **Company** |
| --- | --- | --- | --- |
| hsa-miR-424-5p | CAGCAGCAAUUCAUGUUUUGAA | YP00204736 | Exiqon (Qiagen) |
| hsa-miR-424-3p | CAAAACGUGAGGCGCUGCUAU | YP00205918 | Exiqon (Qiagen) |
| hsa-miR-450a-5p | UUUUGCGAUGUGUUCCUAAUAU | YP00206085 | Exiqon (Qiagen) |
| hsa-miR-450b-5p | UUUUGCAAUAUGUUCCUGAAUA | YP00205607 | Exiqon (Qiagen) |
| hsa-miR-451a-5p | AAACCGUUACCAUUACUGAGUU | YP02119305 | Exiqon (Qiagen) |
| hsa-miR-144-5p | GGAUAUCAUCAUAUACUGUAAG | YP00204670 | Exiqon (Qiagen) |

**Supplemental table 3: Tumor stage and site of metastasis.**

|  | **Non-cachexia**  **(n=11)** | **Cachexia**  **(n= 15)** |
| --- | --- | --- |
| **Tumor stage (n)** |  |  |
| IIIB | 4 | 4 |
| IV | 7 | 11 |
| **Site of metastasis (n)** |  |  |
| Multiple intrapulmonary tumors (both lungs) | 2 | 2 |
| Bone | 1 | 4 |
| Liver | 0 | 1 |
| Lymph nodes | 2 | 1 |
| Peritoneal carcinomatosis | 1 | 0 |
| Pleurisy carcinomatosis | 1 | 3 |

**Supplemental table 4: Clinical and demographic data of NSCC patients with cachexia and controls subjected to TaqMan® miRNA array**

|  | *Healthy Control*  *(n=8)* | *Cachexia*  *(n=8)* |
| --- | --- | --- |
| **Gender (male/female)** | 6/2 | 6/2 |
| **Age (years)** | 61.9 ± 6.7 | 58.3 ± 8.6 |
| **Height (m)** | 1.77 ± 0.1 | 1.74 ± 0.1 |
| **Body weight loss (kg)** | - | 11.2 ± 3.9*** |
| **Body weight loss (%)** | - | 14.2 ± 5.5*** |
| **Body Mass Index (BMI) (kg/m^2^)** | 24.3 ± 2.8 | 26.8 ± 6.1 |
| **Fat Mass Index (FMI) (kg/m^2^)** | 7.0 ± 3.3 | 7.0 ± 3.1 |
| **Fat Free Mass Index (FFMI) (kg/m^2^)** | 18.1 ± 1.1 | 16.3 ± 3.5 |
| **Appendicular Skeletal Muscle Index (ASMI) (kg/m^2^)** | **7.6 ± 0.69** | **5.9 ± 0.84***** |
|  |  |  |
| **IL-6 (pg/ml)** | **41.7 (35. 85)** | **119.3 (62. 354)*** |
| **CRP (mg/l)** | **1.2 (0.5. 1.6)** | **67.8 (26. 188)***** |
|  |  |  |
| **Disease stage of NSCLC (IIIB/IV)** | - | 1/7 |
| **Histology of NSCLC (adeno/squamous cell)** | - | 4/4 |
|  |  |  |
| **Peak torque flexion 60⁰ (Nm)** | 77.8 ± 18.5 | 28.7 ± 18.7*** |
| **Peak torque extension 60⁰ (Nm)** | 119.3 ± 29.6 | 49.8 ± 29.4*** |

*Cachexia was defined as body weight loss of >5% in the past 6 months. or body weight (BW) loss of >2% in combination with either a BMI <20kg/m^2^. or an appendicular skeletal muscle index consistent with sarcopenia determined by DEXA* ***(males<7.26 kg/m^2^; females<5.45 kg/m^2^)****. Data is presented as mean ± SD* ***for normally distributed variables and as median (interquartile range) for variables that were not normally distributed****.* ***Significance was calculated by*** *Independent samples t-test for equality of means (two-tailed) was used testing* ***for normally distributed variables and by Mann Whitney U test for variables that were not normally distributed.*** ** P<0.05 and ***P<0.001.*

**Supplemental table 5: significantly differentially expressed miRNAs in cachectic NSCLC patients compared to healthy controls.**

| **miRNA** | **Normalized miRNA amount relative to healthy control (2^-ΔΔCt^)** | **P-value** |
| --- | --- | --- |
| hsa-miR-450a-5p | 20.3 | 0.006 |
| hsa-miR-424-5p | 20.1 | 0.007 |
| hsa-miR-450b-5p | 16.1 | 0.003 |
| hsa-miR-424-3p | 12.9 | 0.014 |
| hsa-miR-335-3p | 3.9 | 0.049 |
| hsa-miR-103-3p | 0.49 | 0.020 |
| hsa-miR-483-5p | 0.49 | 0.041 |
| hsa-miR-409-3p | 0.46 | 0.020 |
| hsa-miR-15b-5p | 0.41 | 0.036 |
| hsa-miR-370-3p | 0.41 | 0.021 |
| hsa-miR-20a-3p | 0.40 | 0.001 |
| hsa-miR-451a | 0.39 | 0.039 |
| hsa-miR-517c-3p | 0.35 | 0.011 |
| hsa-miR-144-5p | 0.31 | 0.011 |
| hsa-miR-766-3p | 0.27 | 0.008 |
| hsa-miR-1255b | 0.23 | 0.009 |
| hsa-miR-517a-3p | 0.21 | 0.001 |
| hsa-miR-512-3p | 0.19 | 0.000 |
| hsa-miR-522-3p | 0.18 | 0.009 |
| hsa-miR-520g-3p | 0.18 | 0.024 |
| hsa-miR-483-3p | 0.17 | 0.002 |
| hsa-miR-519a-3p | 0.16 | 0.003 |
| hsa-miR-26a-2-3p | 0.13 | 0.018 |
| hsa-miR-485-3p | 0.12 | 0.013 |
| hsa-miR-379-5p | 0.09 | 0.029 |
| hsa-miR-518b | 0.09 | 0.009 |
| hsa-miR-520h | 0.09 | 0.025 |
| hsa-miR-656-3p | 0.05 | 0.000 |

**Supplemental table 6: Genes significantly targeted by the differentially expressed miRNAs**

| **GeneName** | **Species** | **GeneID** |
| --- | --- | --- |
| **DCBLD2** | **Homo sapiens** | **131566** |
| **SMURF1** | **Homo sapiens** | **57154** |
| **IL6R** | **Homo sapiens** | **3570** |
| **NR4A3** | **Homo sapiens** | **8013** |
| **MYC** | **Homo sapiens** | **4609** |
| **ANLN** | **Homo sapiens** | **54443** |
| **WEE1** | **Homo sapiens** | **7465** |
| **ATF6** | **Homo sapiens** | **22926** |
| **CHEK1** | **Homo sapiens** | **1111** |
| **BMPR1A** | **Homo sapiens** | **657** |
| **CDC25A** | **Homo sapiens** | **993** |
| **PTCH1** | **Homo sapiens** | **5727** |
| **KDM5B** | **Homo sapiens** | **10765** |
| **CDC14A** | **Homo sapiens** | **8556** |
| **MAPK3** | **Homo sapiens** | **5595** |
| **SLC40A1** | **Homo sapiens** | **30061** |
| **CCNF** | **Homo sapiens** | **899** |
| **FAM160B2** | **Homo sapiens** | **64760** |
| **CDKN1A** | **Homo sapiens** | **1026** |
| **CAB39** | **Homo sapiens** | **51719** |
| **SRF** | **Homo sapiens** | **6722** |
| **IKBKB** | **Homo sapiens** | **3551** |
| **PTK2B** | **Homo sapiens** | **2185** |
| **TNIP1** | **Homo sapiens** | **10318** |
| **FOXN1** | **Homo sapiens** | **8456** |
| **KIF23** | **Homo sapiens** | **9493** |
| **FASN** | **Homo sapiens** | **2194** |
| **CUL2** | **Homo sapiens** | **8453** |
| **SPI1** | **Homo sapiens** | **6688** |
| **HIF1A** | **Homo sapiens** | **3091** |
| **SIAH1** | **Homo sapiens** | **6477** |
| **MYB** | **Homo sapiens** | **4602** |
| **MMP2** | **Homo sapiens** | **4313** |
| **ABCB1** | **Homo sapiens** | **5243** |
| **NFYB** | **Homo sapiens** | **4801** |
| **CPT1A** | **Homo sapiens** | **1374** |
| **DENND2D** | **Homo sapiens** | **79961** |
| **PTGR1** | **Homo sapiens** | **22949** |
| **OIP5** | **Homo sapiens** | **11339** |
| **CCNE2** | **Homo sapiens** | **9134** |
| **CCNE1** | **Homo sapiens** | **898** |
| **MET** | **Homo sapiens** | **4233** |
| **RHOA** | **Homo sapiens** | **387** |
| **EI24** | **Homo sapiens** | **9538** |
| **GAB1** | **Homo sapiens** | **2549** |
| **ROCK1** | **Homo sapiens** | **6093** |
| **ELF2** | **Homo sapiens** | **1998** |
| **ROCK2** | **Homo sapiens** | **9475** |
| **ALCAM** | **Homo sapiens** | **214** |
| **HNF1A** | **Homo sapiens** | **6927** |
| **HDAC1** | **Homo sapiens** | **3065** |
| **LIN28A** | **Homo sapiens** | **79727** |
| **RASGRF1** | **Homo sapiens** | **5923** |
| **EIF4A1** | **Homo sapiens** | **1973** |
| **IL6** | **Homo sapiens** | **3569** |
| **BBC3** | **Homo sapiens** | **27113** |
| **PBRM1** | **Homo sapiens** | **55193** |
| **KDR** | **Homo sapiens** | **3791** |
| **NOS3** | **Homo sapiens** | **4846** |
| **SMAD4** | **Homo sapiens** | **4089** |
| **CDX2** | **Homo sapiens** | **1045** |
| **HNRNPK** | **Homo sapiens** | **3190** |
| **FUT2** | **Homo sapiens** | **2524** |
| **VEGFA** | **Homo sapiens** | **7422** |
| **MAT1A** | **Homo sapiens** | **4143** |
| **IGF1** | **Homo sapiens** | **3479** |
| **LGALS3** | **Homo sapiens** | **3958** |
| **NFIA** | **Homo sapiens** | **4774** |
| **CTNNB1** | **Homo sapiens** | **1499** |
| **FOXM1** | **Homo sapiens** | **2305** |
| **FOXO1** | **Homo sapiens** | **2308** |
| **RAB5A** | **Homo sapiens** | **5868** |
| **CPNE3** | **Homo sapiens** | **8895** |
| **RECK** | **Homo sapiens** | **8434** |
| **DICER1** | **Homo sapiens** | **23405** |
| **YES1** | **Homo sapiens** | **7525** |
| **MAP3K8** | **Homo sapiens** | **1326** |
| **SOCS6** | **Homo sapiens** | **9306** |
| **BCL2** | **Homo sapiens** | **596** |
| **FGB** | **Homo sapiens** | **2244** |
| **CCND3** | **Homo sapiens** | **896** |
| **CCND1** | **Homo sapiens** | **595** |
| **CKB** | **Homo sapiens** | **1152** |
| **PTEN** | **Homo sapiens** | **5728** |
| **TSC1** | **Homo sapiens** | **7248** |
| **STAG2** | **Homo sapiens** | **10735** |
| **PLAG1** | **Homo sapiens** | **5324** |
| **FRAT1** | **Homo sapiens** | **10023** |
| **SOCS2** | **Homo sapiens** | **8835** |
| **ELAVL1** | **Homo sapiens** | **1994** |
| **SMAD7** | **Homo sapiens** | **4092** |
| **PPARGC1A** | **Homo sapiens** | **10891** |
| **TRIM29** | **Homo sapiens** | **23650** |
| **INSR** | **Homo sapiens** | **3643** |
| **FOXF2** | **Homo sapiens** | **2295** |
| **PURA** | **Homo sapiens** | **5813** |
| **SMO** | **Homo sapiens** | **6608** |
| **MAP2K1** | **Homo sapiens** | **5604** |
| **UGT2B17** | **Homo sapiens** | **7367** |
| **FGFR1** | **Homo sapiens** | **2260** |
| **RSU1** | **Homo sapiens** | **6251** |
| **FXN** | **Homo sapiens** | **2395** |
| **GADD45A** | **Homo sapiens** | **1647** |
| **ADAM10** | **Homo sapiens** | **102** |
| **PTK2** | **Homo sapiens** | **5747** |
| **IL11** | **Homo sapiens** | **3589** |
| **STAT3** | **Homo sapiens** | **6774** |
| **IFNG** | **Homo sapiens** | **3458** |
| **RAP1B** | **Homo sapiens** | **5908** |
| **CDK4** | **Homo sapiens** | **1019** |
| **NLK** | **Homo sapiens** | **51701** |
| **EGR2** | **Homo sapiens** | **1959** |
| **RDX** | **Homo sapiens** | **5962** |
| **TGFBR2** | **Homo sapiens** | **7048** |
| **SOCS3** | **Homo sapiens** | **9021** |
| **TCEAL1** | **Homo sapiens** | **9338** |
| **OXTR** | **Homo sapiens** | **5021** |
| **AXIN2** | **Homo sapiens** | **8313** |
| **PARD3** | **Homo sapiens** | **56288** |
| **ANG** | **Homo sapiens** | **283** |
| **SMAD3** | **Homo sapiens** | **4088** |
| **TIMP1** | **Homo sapiens** | **7076** |
| **AKT1** | **Homo sapiens** | **207** |
| **TMED7** | **Homo sapiens** | **51014** |
| **ZEB1** | **Homo sapiens** | **6935** |
| **RAB14** | **Homo sapiens** | **51552** |
| **MAPK1** | **Homo sapiens** | **5594** |
| **BAX** | **Homo sapiens** | **581** |
| **SMAD2** | **Homo sapiens** | **4087** |
| **TBR1** | **Homo sapiens** | **10716** |
| **TGFB1** | **Homo sapiens** | **7040** |
| **MGMT** | **Homo sapiens** | **4255** |
| **RAB1A** | **Homo sapiens** | **5861** |
| **PHF10** | **Homo sapiens** | **55274** |
| **CDK6** | **Homo sapiens** | **1021** |
| **ESR1** | **Homo sapiens** | **2099** |
| **DLC1** | **Homo sapiens** | **10395** |
| **BID** | **Homo sapiens** | **637** |
| **ABCG2** | **Homo sapiens** | **9429** |
| **MMP9** | **Homo sapiens** | **4318** |
| **PAX6** | **Homo sapiens** | **5080** |
| **MIF** | **Homo sapiens** | **4282** |
| **NTRK3** | **Homo sapiens** | **4916** |
| **TRIM14** | **Homo sapiens** | **9830** |
| **NF1** | **Homo sapiens** | **4763** |
| **STAT1** | **Homo sapiens** | **6772** |
| **MTSS1** | **Homo sapiens** | **9788** |
| **TGIF1** | **Homo sapiens** | **7050** |
| **DAPK2** | **Homo sapiens** | **23604** |
| **PPM1D** | **Homo sapiens** | **8493** |
| **NOTCH3** | **Homo sapiens** | **4854** |
| **SOX2** | **Homo sapiens** | **6657** |
| **YAP1** | **Homo sapiens** | **10413** |
| **MAP3K1** | **Homo sapiens** | **4214** |
| **CDKN2D** | **Homo sapiens** | **1032** |
| **RUNX1** | **Homo sapiens** | **861** |
| **PEBP4** | **Homo sapiens** | **157310** |
| **CTGF** | **Homo sapiens** | **1490** |

**Supplemental table 7a: MiRNA-gene interactions included in the network.**

| **miRNA** | **Genes** | **Source** |
| --- | --- | --- |
| hsa-miR-424-5p | FASN | miRTarBase |
| hsa-miR-424-5p | SMAD3 | miRTarBase |
| hsa-miR-424-5p | VEGFA | miRTarBase |
| hsa-miR-424-5p | SPI1 | miRTarBase |
| hsa-miR-424-5p | HIF1A | miRTarBase |
| hsa-miR-424-5p | SMAD7 | miRTarBase |
| hsa-miR-424-5p | MAP2K1 | miRTarBase |
| hsa-miR-424-5p | FGFR1 | miRTarBase |
| hsa-miR-424-5p | CCND3 | miRTarBase |
| hsa-miR-424-5p | CCND1 | miRTarBase |
| hsa-miR-424-5p | SMURF1 | miRTarBase |
| hsa-miR-450b-5p | BID | miRTarBase |
| hsa-miR-450a-5p | STAT1 | miRTarBase |
| hsa-miR-335-3p | IL6R | miRTarBase |
| hsa-miR-335-3p | NOS3 | miRTarBase |
| hsa-miR-451a | TSC1 | miRTarBase |
| hsa-miR-451a | IKBKB | miRTarBase |
| hsa-miR-451a | RAB14 | miRTarBase |
| hsa-miR-451a | MYC | miRTarBase |
| hsa-miR-451a | AKT1 | miRTarBase |
| hsa-miR-451a | BCL2 | miRTarBase |
| hsa-miR-451a | IL6R | miRTarBase |
| hsa-miR-451a | RAB5A | miRTarBase |
| hsa-miR-451a | CAB39 | miRTarBase |
| hsa-miR-451a | MAPK1 | miRTarBase |
| hsa-miR-409-3p | MET | miRTarBase |
| hsa-miR-409-3p | TGFBR2 | miRTarBase |
| hsa-miR-409-3p | GAB1 | miRTarBase |
| hsa-miR-409-3p | NLK | miRTarBase |
| hsa-miR-409-3p | AKT1 | miRTarBase |
| hsa-miR-409-3p | ZEB1 | miRTarBase |
| hsa-miR-15b-5p | SMAD3 | miRTarBase |
| hsa-miR-15b-5p | KDR | miRTarBase |
| hsa-miR-15b-5p | FASN | miRTarBase |
| hsa-miR-15b-5p | SMAD7 | miRTarBase |
| hsa-miR-15b-5p | BAX | miRTarBase |
| hsa-miR-15b-5p | SMAD2 | miRTarBase |
| hsa-miR-15b-5p | TGFB1 | miRTarBase |
| hsa-miR-15b-5p | CDK4 | miRTarBase |
| hsa-miR-15b-5p | MAPK1 | miRTarBase |
| hsa-miR-15b-5p | VEGFA | miRTarBase |
| hsa-miR-15b-5p | CCND1 | miRTarBase |
| hsa-miR-15b-5p | BCL2 | miRTarBase |
| hsa-miR-15b-5p | SMURF1 | miRTarBase |
| hsa-miR-15b-5p | FOXO1 | miRTarBase |
| hsa-miR-15b-5p | CCND3 | miRTarBase |
| hsa-miR-15b-5p | INSR | miRTarBase |
| hsa-miR-15b-5p | CDKN1A | miRTarBase |
| hsa-miR-20a-3p | PTEN | miRTarBase |
| hsa-miR-20a-3p | BID | miRTarBase |
| hsa-miR-766-3p | CDK4 | miRTarBase |
| hsa-miR-766-3p | MAPK1 | miRTarBase |
| hsa-miR-766-3p | BAX | miRTarBase |
| hsa-miR-144-5p | TGIF1 | miRTarBase |
| hsa-miR-144-5p | MET | miRTarBase |
| hsa-miR-144-5p | ROCK2 | miRTarBase |
| hsa-miR-144-5p | SMAD4 | miRTarBase |
| hsa-miR-370-3p | CTNNB1 | miRTarBase |
| hsa-miR-370-3p | CDKN1A | miRTarBase |
| hsa-miR-370-3p | TGFBR2 | miRTarBase |
| hsa-miR-370-3p | CPT1A | miRTarBase |
| hsa-miR-370-3p | MAP3K8 | miRTarBase |
| hsa-miR-370-3p | FOXO1 | miRTarBase |
| hsa-miR-379-5p | PTK2 | miRTarBase |
| hsa-miR-522-3p | ELAVL1 | miRTarBase |
| hsa-miR-483-5p | MAPK3 | miRTarBase |
| hsa-miR-483-5p | SRF | miRTarBase |
| hsa-miR-483-5p | RHOA | miRTarBase |
| hsa-miR-485-3p | PPARGC1A | miRTarBase |
| hsa-miR-483-3p | SRF | miRTarBase |
| hsa-miR-483-3p | CDK4 | miRTarBase |
| hsa-miR-483-3p | SMAD4 | miRTarBase |
| hsa-miR-483-3p | RHOA | miRTarBase |
| hsa-miR-483-3p | IGF1 | miRTarBase |
| hsa-miR-519a-3p | STAT3 | miRTarBase |
| hsa-miR-519a-3p | TIMP1 | miRTarBase |
| hsa-miR-519a-3p | MAPK1 | miRTarBase |
| hsa-miR-519a-3p | PTEN | miRTarBase |
| hsa-miR-519a-3p | CDKN1A | miRTarBase |
| hsa-miR-519a-3p | ELAVL1 | miRTarBase |
| hsa-miR-519a-3p | TGFBR2 | miRTarBase |
| hsa-miR-519a-3p | SMAD4 | miRTarBase |
| hsa-miR-520h | VEGFA | miRTarBase |
| hsa-miR-520h | CTNNB1 | miRTarBase |
| hsa-miR-520h | CPT1A | miRTarBase |
| hsa-miR-520h | HDAC1 | miRTarBase |
| hsa-miR-520h | CDKN1A | miRTarBase |
| hsa-miR-520h | FOXO1 | miRTarBase |
| hsa-miR-656-3p | BMPR1A | miRTarBase |
| hsa-miR-520g-3p | VEGFA | miRTarBase |
| hsa-miR-520g-3p | CTNNB1 | miRTarBase |
| hsa-miR-520g-3p | CPT1A | miRTarBase |
| hsa-miR-520g-3p | FOXO1 | miRTarBase |
| hsa-miR-520g-3p | SMAD7 | miRTarBase |

**Supplemental table 7b: Pathway-gene interactions included in the network.**

| **Pathways** | **Genes** | **Source** |
| --- | --- | --- |
| Cytokines and Inflammatory Response | TGFB1 | WikiPathways |
| Amyotrophic lateral sclerosis (ALS) | BCL2 | WikiPathways |
| Amyotrophic lateral sclerosis (ALS) | BID | WikiPathways |
| Amyotrophic lateral sclerosis (ALS) | BAX | WikiPathways |
| Amyotrophic lateral sclerosis (ALS) | RAB5A | WikiPathways |
| Type II interferon signaling (IFNG) | STAT1 | WikiPathways |
| Type II interferon signaling (IFNG) | SPI1 | WikiPathways |
| AMP-activated Protein Kinase (AMPK) Signaling | TSC1 | WikiPathways |
| AMP-activated Protein Kinase (AMPK) Signaling | AKT1 | WikiPathways |
| AMP-activated Protein Kinase (AMPK) Signaling | ELAVL1 | WikiPathways |
| AMP-activated Protein Kinase (AMPK) Signaling | CDKN1A | WikiPathways |
| AMP-activated Protein Kinase (AMPK) Signaling | CPT1A | WikiPathways |
| AMP-activated Protein Kinase (AMPK) Signaling | FASN | WikiPathways |
| AMP-activated Protein Kinase (AMPK) Signaling | CAB39 | WikiPathways |
| AMP-activated Protein Kinase (AMPK) Signaling | INSR | WikiPathways |
| IL-6 signaling pathway | MAP2K1 | WikiPathways |
| IL-6 signaling pathway | NLK | WikiPathways |
| IL-6 signaling pathway | HDAC1 | WikiPathways |
| IL-6 signaling pathway | MAPK3 | WikiPathways |
| IL-6 signaling pathway | IL6R | WikiPathways |
| IL-6 signaling pathway | MAPK1 | WikiPathways |
| IL-6 signaling pathway | TIMP1 | WikiPathways |
| IL-6 signaling pathway | STAT3 | WikiPathways |
| IL-6 signaling pathway | AKT1 | WikiPathways |
| IL-6 signaling pathway | STAT1 | WikiPathways |
| IL-6 signaling pathway | GAB1 | WikiPathways |
| TGF beta signaling | TGFB1 | WikiPathways |
| TGF beta signaling | MAPK1 | WikiPathways |
| TGF beta signaling | TGFBR2 | WikiPathways |
| TGF beta signaling | AKT1 | WikiPathways |
| TGF beta signaling | MET | WikiPathways |
| TGF beta signaling | SMAD4 | WikiPathways |
| TGF beta signaling | PTK2 | WikiPathways |
| TGF beta signaling | CDKN1A | WikiPathways |
| TGF beta signaling | MAP2K1 | WikiPathways |
| TGF beta signaling | YAP1 | WikiPathways |
| TGF beta signaling | SMAD3 | WikiPathways |
| TGF beta signaling | SMAD2 | WikiPathways |
| TGF beta signaling | TGIF1 | WikiPathways |
| TGF beta signaling | MYC | WikiPathways |
| TGF beta signaling | HDAC1 | WikiPathways |
| TGF beta signaling | ZEB1 | WikiPathways |
| TGF beta signaling | CCND1 | WikiPathways |
| TGF beta signaling | SMAD7 | WikiPathways |
| TGF beta signaling | RHOA | WikiPathways |
| TGF beta signaling | SMURF1 | WikiPathways |
| TGF beta signaling | CTNNB1 | WikiPathways |
| TGF beta signaling | STAT1 | WikiPathways |
| TGF beta signaling | MAPK3 | WikiPathways |
| TGF beta signaling | STAT3 | WikiPathways |
| TGF beta signaling | BMPR1A | WikiPathways |
| TNFalpha_IL1 signaling | MAPK1 | WikiPathways |
| TNFalpha_IL1 signaling | CTNNB1 | WikiPathways |
| TNFalpha_IL1 signaling | IKBKB | WikiPathways |
| TNFalpha_IL1 signaling | AKT1 | WikiPathways |
| TNFalpha_IL1 signaling | MAP3K8 | WikiPathways |
| TNFalpha_IL1 signaling | MAPK3 | WikiPathways |
| TNFalpha_IL1 signaling | BID | WikiPathways |
| TNFalpha_IL1 signaling | BAX | WikiPathways |
| TNFalpha_IL1 signaling | MAP2K1 | WikiPathways |
| TNFalpha_IL1 signaling | HDAC1 | WikiPathways |
| Insulin signaling | IGF1 | WikiPathways |
| Insulin signaling | MAP3K8 | WikiPathways |
| Insulin signaling | PTEN | WikiPathways |
| Insulin signaling | INSR | WikiPathways |
| Insulin signaling | IKBKB | WikiPathways |
| Insulin signaling | MAP2K1 | WikiPathways |
| Insulin signaling | FOXO1 | WikiPathways |
| Insulin signaling | MAPK1 | WikiPathways |
| Insulin signaling | AKT1 | WikiPathways |
| Insulin signaling | SRF | WikiPathways |
| Insulin signaling | TSC1 | WikiPathways |
| Insulin signaling | MAPK3 | WikiPathways |
| Insulin signaling | GAB1 | WikiPathways |
| Insulin signaling | SMAD3 | WikiPathways |
| Insulin signaling | SMAD2 | WikiPathways |
| Insulin signaling | PPARGC1A | WikiPathways |
| PI3K-Akt-mTOR signaling | AKT1 | WikiPathways |
| PI3K-Akt-mTOR signaling | MET | WikiPathways |
| PI3K-Akt-mTOR signaling | FGFR1 | WikiPathways |
| PI3K-Akt-mTOR signaling | MAPK1 | WikiPathways |
| PI3K-Akt-mTOR signaling | VEGFA | WikiPathways |
| PI3K-Akt-mTOR signaling | KDR | WikiPathways |
| PI3K-Akt-mTOR signaling | NOS3 | WikiPathways |
| PI3K-Akt-mTOR signaling | CCND1 | WikiPathways |
| PI3K-Akt-mTOR signaling | IL6R | WikiPathways |
| PI3K-Akt-mTOR signaling | TSC1 | WikiPathways |
| PI3K-Akt-mTOR signaling | CCND3 | WikiPathways |
| PI3K-Akt-mTOR signaling | INSR | WikiPathways |
| PI3K-Akt-mTOR signaling | MAP2K1 | WikiPathways |
| PI3K-Akt-mTOR signaling | PTK2 | WikiPathways |
| PI3K-Akt-mTOR signaling | IKBKB | WikiPathways |
| PI3K-Akt-mTOR signaling | IGF1 | WikiPathways |
| PI3K-Akt-mTOR signaling | CDKN1A | WikiPathways |
| PI3K-Akt-mTOR signaling | MYC | WikiPathways |
| PI3K-Akt-mTOR signaling | BCL2 | WikiPathways |
| PI3K-Akt-mTOR signaling | MAPK3 | WikiPathways |
| PI3K-Akt-mTOR signaling | CDK4 | WikiPathways |
| PI3K-Akt-mTOR signaling | HIF1A | WikiPathways |
| PI3K-Akt-mTOR signaling | ELAVL1 | WikiPathways |
| PI3K-Akt-mTOR signaling | CAB39 | WikiPathways |
| PI3K-Akt-mTOR signaling | PTEN | WikiPathways |
| PI3K-Akt-mTOR signaling | FOXO1 | WikiPathways |
| PI3K-Akt-mTOR signaling | PPARGC1A | WikiPathways |
| PI3K-Akt-mTOR signaling | RAB14 | WikiPathways |
| Cardiac related pathways | TGFB1 | WikiPathways |
| Cardiac related pathways | MAPK1 | WikiPathways |
| Cardiac related pathways | IKBKB | WikiPathways |
| Cardiac related pathways | IGF1 | WikiPathways |
| Cardiac related pathways | MAP2K1 | WikiPathways |
| Cardiac related pathways | MAPK3 | WikiPathways |
| Cardiac related pathways | KDR | WikiPathways |
| Cardiac related pathways | STAT3 | WikiPathways |
| Cardiac related pathways | RHOA | WikiPathways |
| Cardiac related pathways | AKT1 | WikiPathways |
| Cardiac related pathways | ROCK2 | WikiPathways |
| Cardiac related pathways | CTNNB1 | WikiPathways |

**Supplemental table 8: Pathways significantly targeted by the differentially expressed miRNAs**

| **Pathway** | **positive (r)** | **measured (n)** | **total** | **%** | **Z Score** | **p-value (permuted)** |
| --- | --- | --- | --- | --- | --- | --- |
| AGE/RAGE pathway | 18 | 66 | 67 | 27.27% | 10.79 | 0 |
| Chromosomal and microsatellite instability in colorectal cancer | 19 | 73 | 81 | 26.03% | 10.76 | 0 |
| Integrated Breast Cancer Pathway | 30 | 164 | 201 | 18.29% | 10.68 | 0 |
| Pancreatic adenocarcinoma pathway | 20 | 89 | 97 | 22.47% | 10.03 | 0 |
| Leptin signaling pathway | 18 | 75 | 77 | 24.00% | 9.93 | 0 |
| IL-6 signaling pathway | 13 | 43 | 45 | 30.23% | 9.78 | 0 |
| Hepatitis C and Hepatocellular Carcinoma | 14 | 50 | 63 | 28.00% | 9.67 | 0 |
| Integrated Cancer Pathway | 13 | 45 | 50 | 28.89% | 9.5 | 0 |
| IL-7 Signaling Pathway | 9 | 25 | 26 | 36.00% | 9.05 | 0 |
| Interleukin-11 Signaling Pathway | 12 | 44 | 45 | 27.27% | 8.8 | 0 |
| Signaling of Hepatocyte Growth Factor Receptor | 10 | 34 | 35 | 29.41% | 8.42 | 0 |
| TGF-B Signaling in Thyroid Cells for Epithelial-Mesenchymal Transition | 7 | 18 | 20 | 38.89% | 8.35 | 0 |
| Angiogenesis | 8 | 24 | 25 | 33.33% | 8.14 | 0 |
| Cell Cycle | 20 | 120 | 124 | 16.67% | 8.12 | 0 |
| Signaling Pathways in Glioblastoma | 16 | 83 | 88 | 19.28% | 8.06 | 0 |
| TGF-beta Signaling Pathway | 21 | 132 | 133 | 15.91% | 8.04 | 0 |
| DNA Damage Response (only ATM dependent) | 19 | 113 | 118 | 16.81% | 7.96 | 0 |
| DNA Damage Response | 14 | 68 | 76 | 20.59% | 7.89 | 0 |
| Androgen receptor signaling pathway | 16 | 89 | 90 | 17.98% | 7.67 | 0 |
| Endometrial cancer | 13 | 63 | 75 | 20.63% | 7.61 | 0 |
| TP53 Network | 7 | 21 | 23 | 33.33% | 7.61 | 0 |
| TGF-beta Receptor Signaling | 12 | 55 | 56 | 21.82% | 7.6 | 0 |
| Bladder Cancer | 10 | 40 | 47 | 25.00% | 7.59 | 0 |
| Hypoxia-mediated EMT and Stemness | 2 | 2 | 6 | 100.00% | 7.57 | 0 |
| IL-2 Signaling Pathway | 10 | 42 | 43 | 23.81% | 7.35 | 0 |
| VEGFA-VEGFR2 Signaling Pathway | 28 | 236 | 238 | 11.86% | 7.32 | 0 |
| Canonical and Non-Canonical TGF-B signaling | 6 | 17 | 18 | 35.29% | 7.3 | 0 |
| RAC1/PAK1/p38/MMP2 Pathway | 13 | 68 | 74 | 19.12% | 7.22 | 0 |
| Wnt Signaling Pathway | 11 | 51 | 52 | 21.57% | 7.22 | 0 |
| Extracellular vesicle-mediated signaling in recipient cells | 8 | 30 | 31 | 26.67% | 7.08 | 0 |
| TCA Cycle Nutrient Utilization and Invasiveness of Ovarian Cancer | 3 | 5 | 11 | 60.00% | 7.01 | 0 |
| Osteopontin Signaling | 5 | 13 | 14 | 38.46% | 7.01 | 0 |
| Hypothesized Pathways in Pathogenesis of Cardiovascular Disease | 7 | 25 | 27 | 28.00% | 6.83 | 0 |
| G1 to S cell cycle control | 12 | 65 | 66 | 18.46% | 6.76 | 0 |
| miRNA Regulation of DNA Damage Response | 14 | 87 | 106 | 16.09% | 6.6 | 0 |
| Somatroph axis (GH) and its relationship to dietary restriction and aging | 3 | 6 | 12 | 50.00% | 6.33 | 0 |
| Extracellular vesicles in the crosstalk of cardiac cells | 6 | 22 | 30 | 27.27% | 6.22 | 0 |
| Breast cancer pathway | 19 | 156 | 167 | 12.18% | 6.15 | 0 |
| Viral Acute Myocarditis | 13 | 85 | 109 | 15.29% | 6.12 | 0 |
| Estrogen signaling pathway | 6 | 23 | 26 | 26.09% | 6.04 | 0 |
| Thymic Stromal LymphoPoietin (TSLP) Signaling Pathway | 9 | 47 | 49 | 19.15% | 6.01 | 0 |
| Human Thyroid Stimulating Hormone (TSH) signaling pathway | 11 | 66 | 67 | 16.67% | 6 | 0 |
| Non-small cell lung cancer | 11 | 66 | 76 | 16.67% | 6 | 0 |
| Retinoblastoma Gene in Cancer | 13 | 88 | 98 | 14.77% | 5.95 | 0 |
| IL-9 Signaling Pathway | 5 | 17 | 18 | 29.41% | 5.95 | 0 |
| Leptin Insulin Overlap | 5 | 17 | 20 | 29.41% | 5.95 | 0 |
| PDGF Pathway | 8 | 39 | 52 | 20.51% | 5.94 | 0 |
| IL-5 Signaling Pathway | 8 | 40 | 41 | 20.00% | 5.84 | 0 |
| 4-hydroxytamoxifen. Dexamethasone. and Retinoic Acids Regulation of p27 Expression | 5 | 18 | 31 | 27.78% | 5.74 | 0 |
| ncRNAs involved in STAT3 signaling in hepatocellular carcinoma | 5 | 18 | 19 | 27.78% | 5.74 | 0 |
| Prion disease pathway | 7 | 33 | 36 | 21.21% | 5.69 | 0 |
| Adipogenesis | 16 | 130 | 132 | 12.31% | 5.68 | 0 |
| TNF related weak inducer of apoptosis (TWEAK) Signaling Pathway | 8 | 42 | 45 | 19.05% | 5.64 | 0 |
| EPO Receptor Signaling | 6 | 26 | 27 | 23.08% | 5.57 | 0 |
| Overview of nanoparticle effects | 5 | 19 | 41 | 26.32% | 5.54 | 0 |
| Endochondral Ossification | 10 | 63 | 69 | 15.87% | 5.51 | 0 |
| TFs Regulate miRNAs related to cardiac hypertrophy | 4 | 13 | 16 | 30.77% | 5.47 | 0 |
| Oncostatin M Signaling Pathway | 10 | 65 | 66 | 15.38% | 5.38 | 0 |
| Hypertrophy Model | 5 | 20 | 21 | 25.00% | 5.36 | 0.001 |
| Serotonin Receptor 2 and ELK-SRF/GATA4 signaling | 5 | 20 | 24 | 25.00% | 5.36 | 0 |
| Caloric restriction and aging | 3 | 8 | 13 | 37.50% | 5.35 | 0.001 |
| Spinal Cord Injury | 14 | 116 | 127 | 12.07% | 5.22 | 0 |
| Regulation of Apoptosis by Parathyroid Hormone-related Protein | 5 | 22 | 24 | 22.73% | 5.03 | 0 |
| Factors and pathways affecting insulin-like growth factor (IGF1)-Akt signaling | 6 | 31 | 34 | 19.35% | 4.94 | 0 |
| miRNA regulation of p53 pathway in prostate cancer | 6 | 31 | 37 | 19.35% | 4.94 | 0.001 |
| Focal Adhesion | 19 | 198 | 201 | 9.60% | 4.9 | 0 |
| MicroRNAs in cardiomyocyte hypertrophy | 12 | 98 | 109 | 12.24% | 4.89 | 0 |
| Pathways in clear cell renal cell carcinoma | 11 | 86 | 92 | 12.79% | 4.86 | 0 |
| PI3K-Akt Signaling Pathway | 27 | 340 | 359 | 7.94% | 4.76 | 0 |
| Prolactin Signaling Pathway | 10 | 76 | 79 | 13.16% | 4.74 | 0.001 |
| Apoptosis-related network due to altered Notch3 in ovarian cancer | 8 | 53 | 54 | 15.09% | 4.74 | 0 |
| Alpha 6 Beta 4 signaling pathway | 6 | 33 | 34 | 18.18% | 4.72 | 0 |
| Epithelial to mesenchymal transition in colorectal cancer | 16 | 160 | 161 | 10.00% | 4.68 | 0 |
| Cardiac Hypertrophic Response | 8 | 54 | 61 | 14.81% | 4.67 | 0 |
| Association Between Physico-Chemical Features and Toxicity Associated Pathways | 9 | 66 | 78 | 13.64% | 4.63 | 0 |
| ErbB Signaling Pathway | 8 | 55 | 64 | 14.55% | 4.6 | 0 |
| RANKL/RANK (Receptor activator of NFKB (ligand)) Signaling Pathway | 8 | 55 | 58 | 14.55% | 4.6 | 0 |
| Corticotropin-releasing hormone signaling pathway | 11 | 92 | 98 | 11.96% | 4.58 | 0.001 |
| Senescence and Autophagy in Cancer | 12 | 106 | 112 | 11.32% | 4.56 | 0 |
| Photodynamic therapy-induced NF-kB survival signaling | 6 | 35 | 36 | 17.14% | 4.52 | 0.001 |
| Wnt/beta-catenin Signaling Pathway in Leukemia | 5 | 26 | 29 | 19.23% | 4.48 | 0 |
| Aryl Hydrocarbon Receptor | 7 | 47 | 57 | 14.89% | 4.39 | 0.001 |
| Kit receptor signaling pathway | 8 | 59 | 60 | 13.56% | 4.35 | 0.001 |
| Serotonin Receptor 4/6/7 and NR3C Signaling | 4 | 19 | 22 | 21.05% | 4.27 | 0.002 |
| IL-3 Signaling Pathway | 7 | 49 | 50 | 14.29% | 4.24 | 0.001 |
| Notch Signaling Pathway | 8 | 61 | 62 | 13.11% | 4.23 | 0 |
| ESC Pluripotency Pathways | 12 | 115 | 123 | 10.43% | 4.22 | 0.002 |
| Hair Follicle Development: Cytodifferentiation (Part 3 of 3) | 10 | 87 | 92 | 11.49% | 4.22 | 0 |
| Focal Adhesion-PI3K-Akt-mTOR-signaling pathway | 23 | 301 | 305 | 7.64% | 4.17 | 0 |
| Bone Morphogenic Protein (BMP) Signalling and Regulation | 3 | 12 | 13 | 25.00% | 4.15 | 0.003 |
| LncRNA-mediated mechanisms of therapeutic resistance | 3 | 12 | 14 | 25.00% | 4.15 | 0.004 |
| MAPK and NFkB Signalling Pathways Inhibited by Yersinia YopJ | 3 | 12 | 13 | 25.00% | 4.15 | 0.004 |
| Imatinib and Chronic Myeloid Leukemia | 4 | 20 | 25 | 20.00% | 4.12 | 0.001 |
| Lung fibrosis | 8 | 63 | 85 | 12.70% | 4.11 | 0 |
| Robo4 and VEGF Signaling Pathways Crosstalk | 2 | 6 | 7 | 33.33% | 4.07 | 0.005 |
| miRNAs involved in DNA damage response | 7 | 52 | 70 | 13.46% | 4.04 | 0 |
| ATM Signaling Pathway | 6 | 41 | 50 | 14.63% | 4 | 0.001 |
| IL17 signaling pathway | 5 | 31 | 32 | 16.13% | 3.94 | 0.001 |
| MFAP5-mediated ovarian cancer cell motility and invasiveness | 3 | 13 | 18 | 23.08% | 3.94 | 0.003 |
| IL-4 Signaling Pathway | 7 | 55 | 56 | 12.73% | 3.85 | 0.002 |
| Transcription factor regulation in adipogenesis | 4 | 22 | 24 | 18.18% | 3.85 | 0.002 |
| AMP-activated Protein Kinase (AMPK) Signaling | 8 | 68 | 78 | 11.76% | 3.85 | 0.002 |
| Brain-Derived Neurotrophic Factor (BDNF) signaling pathway | 13 | 144 | 150 | 9.03% | 3.79 | 0.001 |
| Insulin Signaling | 14 | 161 | 162 | 8.70% | 3.77 | 0 |
| BDNF-TrkB Signaling | 5 | 33 | 38 | 15.15% | 3.75 | 0.001 |
| EGF/EGFR Signaling Pathway | 14 | 162 | 163 | 8.64% | 3.75 | 0.001 |
| Type 2 papillary renal cell carcinoma | 5 | 34 | 41 | 14.71% | 3.67 | 0 |
| Butyrate-induced histone acetylation | 1 | 2 | 11 | 50.00% | 3.65 | 0.005 |
| Influenza A virus infection | 1 | 2 | 16 | 50.00% | 3.65 | 0.006 |
| Heart Development | 6 | 46 | 48 | 13.04% | 3.64 | 0.003 |
| Ras Signaling | 15 | 184 | 192 | 8.15% | 3.63 | 0.002 |
| Apoptosis | 9 | 87 | 88 | 10.34% | 3.62 | 0.001 |
| H19 action Rb-E2F1 signaling and CDK-Beta-catenin activity | 3 | 15 | 17 | 20.00% | 3.57 | 0.007 |
| T-Cell antigen Receptor (TCR) pathway during Staphylococcus aureus infection | 7 | 61 | 70 | 11.48% | 3.52 | 0.002 |
| Structural Pathway of Interleukin 1 (IL-1) | 6 | 48 | 52 | 12.50% | 3.51 | 0.004 |
| T-Cell antigen Receptor (TCR) Signaling Pathway | 9 | 90 | 93 | 10.00% | 3.5 | 0.002 |
| TNF alpha Signaling Pathway | 9 | 92 | 97 | 9.78% | 3.42 | 0.003 |
| Cytokines and Inflammatory Response | 4 | 26 | 32 | 15.38% | 3.4 | 0.009 |
| PPAR Alpha Pathway | 4 | 26 | 28 | 15.38% | 3.4 | 0.007 |
| Differentiation Pathway | 6 | 50 | 64 | 12.00% | 3.39 | 0.004 |
| Follicle Stimulating Hormone (FSH) signaling pathway | 4 | 27 | 28 | 14.81% | 3.3 | 0.005 |
| Chemokine signaling pathway | 13 | 165 | 172 | 7.88% | 3.23 | 0.002 |
| Cardiac Progenitor Differentiation | 6 | 53 | 56 | 11.32% | 3.21 | 0.001 |
| LncRNA involvement in canonical Wnt signaling and colorectal cancer | 9 | 98 | 102 | 9.18% | 3.2 | 0.004 |
| MicroRNA for Targeting Cancer Growth and Vascularization in Glioblastoma | 2 | 9 | 10 | 22.22% | 3.13 | 0.014 |
| Tgif disruption of Shh signaling | 2 | 9 | 10 | 22.22% | 3.13 | 0.023 |
| Inhibition of exosome biogenesis and secretion by Manumycin A in CRPC cells | 3 | 18 | 19 | 16.67% | 3.13 | 0.009 |
| MAPK Cascade | 4 | 29 | 33 | 13.79% | 3.11 | 0.01 |
| IL-1 signaling pathway | 6 | 55 | 57 | 10.91% | 3.1 | 0.005 |
| Wnt Signaling Pathway and Pluripotency | 9 | 101 | 106 | 8.91% | 3.1 | 0.003 |
| Mesodermal Commitment Pathway | 12 | 153 | 155 | 7.84% | 3.09 | 0.003 |
| MAPK Signaling Pathway | 17 | 249 | 259 | 6.83% | 3.06 | 0.011 |
| PI3K-AKT-mTOR signaling pathway and therapeutic opportunities | 4 | 30 | 33 | 13.33% | 3.03 | 0.014 |
| Hedgehog Signaling Pathway | 5 | 43 | 44 | 11.63% | 3 | 0.006 |
| Tumor suppressor activity of SMARCB1 | 4 | 31 | 35 | 12.90% | 2.94 | 0.013 |
| BMP Signaling Pathway in Eyelid Development | 3 | 20 | 23 | 15.00% | 2.88 | 0.014 |
| Hematopoietic Stem Cell Gene Regulation by GABP alpha/beta Complex | 3 | 20 | 26 | 15.00% | 2.88 | 0.015 |
| Nanomaterial induced apoptosis | 3 | 20 | 30 | 15.00% | 2.88 | 0.007 |
| Endoderm Differentiation | 11 | 144 | 147 | 7.64% | 2.86 | 0.012 |
| NAD metabolism. sirtuins and aging | 2 | 11 | 16 | 18.18% | 2.72 | 0.01 |
| p38 MAPK Signaling Pathway | 4 | 34 | 36 | 11.76% | 2.71 | 0.013 |
| PI3K/AKT/mTOR - VitD3 Signalling | 3 | 22 | 34 | 13.64% | 2.67 | 0.01 |
| Type II diabetes mellitus | 3 | 22 | 28 | 13.64% | 2.67 | 0.017 |
| Angiopoietin Like Protein 8 Regulatory Pathway | 10 | 133 | 156 | 7.52% | 2.67 | 0.008 |
| Nonalcoholic fatty liver disease | 11 | 155 | 170 | 7.10% | 2.59 | 0.015 |
| Nanoparticle triggered autophagic cell death | 3 | 23 | 29 | 13.04% | 2.57 | 0.029 |
| Toll-like Receptor Signaling Pathway | 8 | 102 | 109 | 7.84% | 2.51 | 0.017 |
| Fibrin Complement Receptor 3 Signaling Pathway | 4 | 37 | 44 | 10.81% | 2.51 | 0.016 |
| Photodynamic therapy-induced HIF-1 survival signaling | 4 | 37 | 38 | 10.81% | 2.51 | 0.015 |
| Type II interferon signaling (IFNG) | 4 | 37 | 38 | 10.81% | 2.51 | 0.014 |
| IL1 and megakaryocytes in obesity | 3 | 24 | 26 | 12.50% | 2.48 | 0.03 |
| Physiological and Pathological Hypertrophy of the Heart | 3 | 24 | 27 | 12.50% | 2.48 | 0.023 |
| Amyotrophic lateral sclerosis (ALS) | 4 | 38 | 56 | 10.53% | 2.45 | 0.009 |
| ncRNAs involved in Wnt signaling in hepatocellular carcinoma | 7 | 87 | 89 | 8.05% | 2.43 | 0.021 |
| Vitamin D Receptor Pathway | 12 | 183 | 188 | 6.56% | 2.41 | 0.019 |
| Copper homeostasis | 5 | 54 | 58 | 9.26% | 2.4 | 0.014 |
| Interferon type I signaling pathways | 5 | 54 | 57 | 9.26% | 2.4 | 0.016 |
| BMP2-WNT4-FOXO1 Pathway in Human Primary Endometrial Stromal Cell Differentiation | 2 | 13 | 16 | 15.38% | 2.4 | 0.031 |
| Development of pulmonary dendritic cells and macrophage subsets | 2 | 13 | 14 | 15.38% | 2.4 | 0.033 |
| Estrogen Receptor Pathway | 2 | 13 | 16 | 15.38% | 2.4 | 0.034 |
| Oxytocin signaling | 1 | 4 | 10 | 25.00% | 2.4 | 0.015 |
| Serotonin Receptor 2 and STAT3 Signaling | 1 | 4 | 6 | 25.00% | 2.4 | 0.025 |
| Signal Transduction of S1P Receptor | 3 | 25 | 26 | 12.00% | 2.39 | 0.018 |
| Non-genomic actions of 1.25 dihydroxyvitamin D3 | 6 | 71 | 97 | 8.45% | 2.38 | 0.024 |
| Sudden Infant Death Syndrome (SIDS) Susceptibility Pathways | 11 | 166 | 182 | 6.63% | 2.34 | 0.016 |
| Oxidative Damage | 4 | 40 | 44 | 10.00% | 2.33 | 0.019 |
| Common Pathways Underlying Drug Addiction | 4 | 41 | 50 | 9.76% | 2.27 | 0.037 |
| Nanoparticle-mediated activation of receptor signaling | 3 | 28 | 36 | 10.71% | 2.15 | 0.035 |
| let-7 inhibition of ES cell reprogramming | 2 | 15 | 17 | 13.33% | 2.14 | 0.057 |
| Wnt Signaling Pathway | 8 | 116 | 121 | 6.90% | 2.11 | 0.027 |
| B Cell Receptor Signaling Pathway | 7 | 97 | 99 | 7.22% | 2.11 | 0.037 |
| miR-517 relationship with ARCN1 and USP1 | 1 | 5 | 7 | 20.00% | 2.06 | 0.037 |
| RNA interference | 1 | 5 | 6 | 20.00% | 2.06 | 0.066 |
| Ultraconserved region 339 modulation of tumor suppressor microRNAs in cancer | 1 | 5 | 6 | 20.00% | 2.06 | 0.056 |
| Hedgehog Signaling Pathway | 2 | 16 | 17 | 12.50% | 2.02 | 0.061 |
| Neural Crest Differentiation | 7 | 100 | 102 | 7.00% | 2.02 | 0.037 |
| Dopaminergic Neurogenesis | 3 | 30 | 32 | 10.00% | 2.01 | 0.051 |
| Matrix Metalloproteinases | 3 | 30 | 31 | 10.00% | 2.01 | 0.043 |
| Regulation of Microtubule Cytoskeleton | 4 | 46 | 47 | 8.70% | 2 | 0.037 |
| Integrin-mediated Cell Adhesion | 7 | 101 | 102 | 6.93% | 1.99 | 0.042 |
| Ectoderm Differentiation | 9 | 142 | 145 | 6.34% | 1.97 | 0.037 |

***N: measured data points; R: data points meeting criterion.***

***The pathways were ranked based on a Z score. A pathway was considered involved when the Z score > 1.96 and permutation p value < 0.05.*** *Criterion: [positive] = 1. Calculation method: pathway-centric.*
